# Acute measurement of flow‐mediated dilation following passive heating in adults: The confounding role of altered shear stress and baseline vasodilation

**DOI:** 10.14814/phy2.70723

**Published:** 2026-02-16

**Authors:** Campbell Menzies, Neil D. Clarke, Charles J. Steward, C. Douglas Thake, Christopher J. A. Pugh, Tom Cullen

**Affiliations:** ^1^ Centre for Physical Activity, Sport & Exercise Sciences Coventry University Coventry UK; ^2^ School of Life Sciences University of Nottingham Nottingham UK; ^3^ College of Life Sciences, Faculty of Health, Education and Life Sciences Birmingham City University Birmingham UK; ^4^ Cardiff School of Sport & Health Sciences Cardiff Metropolitan University Cardiff UK; ^5^ Centre for Cardiovascular Research, Innovation and Development Cardiff Metropolitan University Cardiff UK

**Keywords:** FMD, LFMC, methodology, passive heating, vascular function

## Abstract

Changes in flow‐mediated dilation (FMD) following acute heating are not well understood, appear protocol‐specific, and may be better understood by additional measures of acute vasoactivity. This study investigated FMD responses before and after three different 30‐min hot‐water immersion conditions (40°C‐Shoulder, 42°C‐Waist, and 40°C‐Waist) in 22 adults. Brachial artery diameter was recorded at baseline (D_base_), during the final 30 s of occlusion (D_occ_), and at peak post‐occlusion (D_peak_). Allometrically scaled FMD%, and changes in diameter during occlusion (OIV), and from end‐occlusion to peak diameter (FMD_Docc_) were calculated. Pre‐occlusion shear rate was greater post‐immersion in 40‐Shoulder (*p* < 0.001) and 42‐Waist (p < 0.001), but not 40‐Waist (*p* = 0.13), with the largest increase observed in 40‐Shoulder. Alongside this, D_base_ increased (Δ0.4 ± 0.2 mm, p < 0.001) and FMD% decreased (Δ−3.9 ± 3.8%, *p* = 0.04) following immersion in 40°C‐Shoulder only. Across all conditions, ΔFMD% was negatively associated with ΔD_base_ (*r*_rm_ = −0.47, *p* = 0.001). OIV% was the only vasoactivity metric to statistically differentiate between all conditions post‐immersion (40°C‐Shoulder: −8.1 ± 4.9%. 42°C‐Waist: −3.0 ± 5.3%. 40°C‐Waist: 1.1 ± 4.1%. *p* < 0.001). Post‐heating FMD is confounded by heat‐induced increases in baseline diameter, even after allometric scaling, while OIV% may provide complementary insight into acute vasoactivity following passive heating.

## 1 INTRODUCTION

Flow‐mediated dilation (FMD) is a noninvasive measure of peripheral artery endothelium‐dependent dilation (Thijssen et al., 2019) stimulated by the surge in shear stress following occlusion (Pyke & Tschakovsky, 2007) and is calculated as the difference between baseline and peak post‐occlusion arterial diameter. Increased FMD at rest represents improved endothelial function and is linked to lower risk of cardiovascular events (Inaba et al., 2010), supporting its use as a prognostic marker in interventional studies (Thijssen et al., 2019). Acute physiological stimuli such as passive heating or exercise can alter FMD, and these acute responses may relate to chronic adaptation with repeated exposures (Dawson et al., 2018; Romero et al., 2022). However, passive heating appears to elicit varied acute FMD responses, with some studies reporting increases (Coombs et al., 2021; Tinken et al., 2009) and others decreases (Alali et al., 2020; Chaseling et al., 2023), which may, in part, be attributable to the specific heating stimulus. Although a transient reduction in FMD might be interpreted as an acute endothelial impairment, this does not reflect the beneficial adaptive endothelial response observed with repeated heat exposures (Bailey et al., 2016; Brunt et al., 2016; Carter et al., 2014; Steward et al., 2025). Accordingly, interpretation of these acute results requires careful consideration within the context of the specific acute stimuli influencing the FMD response.

The differences in acute FMD responses following passive heating may be explained by the specific effects of the heating protocol preceding the FMD measurement. FMD is directly influenced by baseline arterial diameter (Atkinson & Batterham, 2013) and/or preceding antegrade or retrograde shear stress patterns (Green et al., 2017). This well‐established inverse relationship between baseline (i.e., pre‐occlusion) arterial diameter and FMD under resting conditions has led to recommendations that FMD be allometrically scaled for baseline diameter to enable independent inference from diameter and FMD (Atkinson & Batterham, 2013). Acute changes in FMD following heating protocols may also differ according to the magnitude of shear stress. Large sustained increases in antegrade shear rate, often observed with prolonged or larger heating stimuli, elicit baseline vasodilation and partially utilize a vessel's vasodilatory reserve, thereby attenuating the subsequent FMD response (Alali et al., 2020; Chaseling et al., 2023; Coombs et al., 2021). In contrast, smaller increases in antegrade shear rate, as typically seen with local heating protocols, may act to “prime” the endothelium and enhance the subsequent FMD response (Thijssen et al., 2014; Tinken et al., 2009). Therefore, the comparative confounding effects of shear rate and increased baseline diameter on FMD interpretation are likely context specific and will vary between heating protocols and other acute physiological stimuli (e.g., exercise).

Acute increases in baseline brachial artery diameter following exercise have been previously identified as problematic in the interpretation of acute FMD responses (Padilla et al., 2007). To minimize the influence of heating‐induced increases in shear rate on baseline diameter, some authors have replaced traditional pre‐occlusion diameter with the arterial diameter in the final 30 s of occlusion (Chaseling et al., 2023; Coombs et al., 2021). However, the physiological relevance of this modified FMD calculation remains uncertain, as it is unclear whether it can be interpreted in the same manner as “traditional” FMD calculations (Chaseling et al., 2023). Alternatively, this modified FMD metric, alongside the change in diameter during occlusion (often termed low flow‐mediated constriction LFMC), may offer complementary insight into the endothelial environment (Humphreys et al., 2014). Marked reductions in arterial diameter have been observed during occlusion immediately following heating (Alali et al., 2020; Chaseling et al., 2023), which is consistent with observations showing LFMC is greater in vessels with larger baseline diameters (Sen et al., 2020). However, this response may reflect the withdrawal of the heating (and shear stress) stimulus rather than active vasoconstriction per se, meaning that the term LFMC may be misleading in the context of post‐heating arterial vasoactivity. Nevertheless, these vascular alterations may be central to interpreting acute endothelial function and the subsequent FMD response, meaning consideration of these additional metrics may help avoid misinterpretation of acute post‐heating decreases in FMD as endothelial impairment.

We previously demonstrated distinct region‐specific arterial haemodynamic effects in response to 30‐min hot water immersion protocols of differing immersion depth and water temperature (Menzies et al., 2025). Specifically, 40°C shoulder‐deep immersion elicited larger brachial artery vasodilation and elevations in shear rate than either 40°C or 42°C waist‐deep immersion. The present paper aimed to investigate the impact of these protocols and effects on the subsequent post‐immersion FMD response. It was hypothesized that greater increases in baseline diameter post‐heating as seen with 40°C shoulder‐deep immersion would result in larger reductions in FMD. A secondary aim was to examine the acute effects of heating on different indices of vasoactivity (e.g., change in arterial diameter from end‐occlusion relative to baseline and to peak post‐occlusion) to investigate whether these metrics complement or help explain acute changes in FMD.

## 2 METHODS

### 2.1 Experimental design

Ethical approval was provided by the Coventry University Ethics committee (P146084) and conformed to the Declaration of Helsinki, except for prior registration in a database. Data collection for this study was conducted as part of a wider investigation examining vascular, inflammatory, and perceptual responses to 30‐min hot water immersion protocols of differing water depth and temperature. However, the present study addresses a distinct a priori hypothesis relating to the acute FMD response. This is reflected in the inclusion of FMD in the a priori sample size calculation, as described within (Menzies et al., 2025), using the acute FMD data from different heating protocols observed within (Chaseling et al., 2023) (*d* = 0.74). Accordingly, 22 healthy adults (Data given as mean ± SD. 13 males: 29 ± 6 years, 80.3 ± 14.4 kg, 1.78 ± 0.09 m, 25.3 ± 4.4 kg/m^2^. 9 females: 27 ± 6 years, 62.1 ± 8.8 kg, 1.64 ± 0.05 m, 22.9 ± 2.2 kg/m^2^) were recruited locally from responses to social media advertisements and through word of mouth, before being briefed and providing written consent to participate in the present study. Participants attended the laboratory at the same time of day (±1 h) on three separate occasions (10 ± 9 days between visits) to complete 30 minutes of hot water immersion consisting of (i) 40°C shoulder‐deep immersion (40‐Shoulder) (40.0 ± 0.1°C), (ii) 42°C waist‐deep immersion (42‐Waist) (42.0 ± 0.1°C), or (iii) 40°C waist‐deep immersion (40‐Waist) (40.0 ± 0.1°C), completed in a randomized crossover design. FMD was performed following at least 10 minutes of supine rest pre‐immersion and commencing 9 min post‐immersion. The post‐immersion time point was chosen to ensure there was no variation in duration between conditions or participants caused during the transfer from the hot tub to the start of the FMD assessment. For the waist‐deep conditions, participants sat on an 18 cm stool without immersion of their arms in a water depth of ~36 cm. In the shoulder‐deep condition, participants sat on the bottom of the hot tub with their arms submerged in a water depth of 47–52 cm, adjusted according to the participants' height, and moved to the stool for ~3 min with non‐submersion of their arms after 5, 15, and 25 min. Water temperature was constantly monitored using a thermistor (Grant instruments, UK) and maintained using a hot tub heating generator (LazySpa Majorca, Bestway International Ltd., Hong Kong) set to 40°C, with addition and removal of hot or cold water as required to maintain temperature and depth.

### 2.2 Vascular imaging

The FMD assessment consisted of a 9‐min recording of the brachial artery in the distal third of the upper right arm and was collected in accordance with international guidelines (Thijssen et al., 2019). This consisted of 1‐min recorded at baseline, followed by a 5‐min period of forearm occlusion achieved via rapid cuff inflation >220 mmHg immediately distal to the olecranon process (Hokason E20, Bellevue, WA) and continued for 3 min after cuff release.

Vascular imaging was performed using a 15‐MHz multifrequency linear array probe attached to a high‐resolution duplex ultrasound machine (T3300; Tersaon, Burlington, MA). Images were taken following optimisation of the longitudinal B‐mode image of the lumen‐arterial interface, with simultaneous Doppler velocity assessments collected using the smallest possible insonation angle (always <60°). Analysis of brachial artery blood flow, diameter, and shear rate was performed using custom‐designed edge‐detection and wall‐tracking software, as previously described elsewhere (Woodman et al., 2001). Changes in these measures throughout immersion protocols at 5, 15, and 25 min are reported elsewhere (Menzies et al., 2025). Blood flow was calculated as lumen cross‐sectional area × Doppler velocity and shear rate (as an estimate of shear stress without viscosity) as four times the mean blood velocity/vessel diameter. The shear rate area under the curve (SR_AUC_) was used to reflect the hyperaemic shear stress stimulus upon cuff release.

**FIGURE 1:**
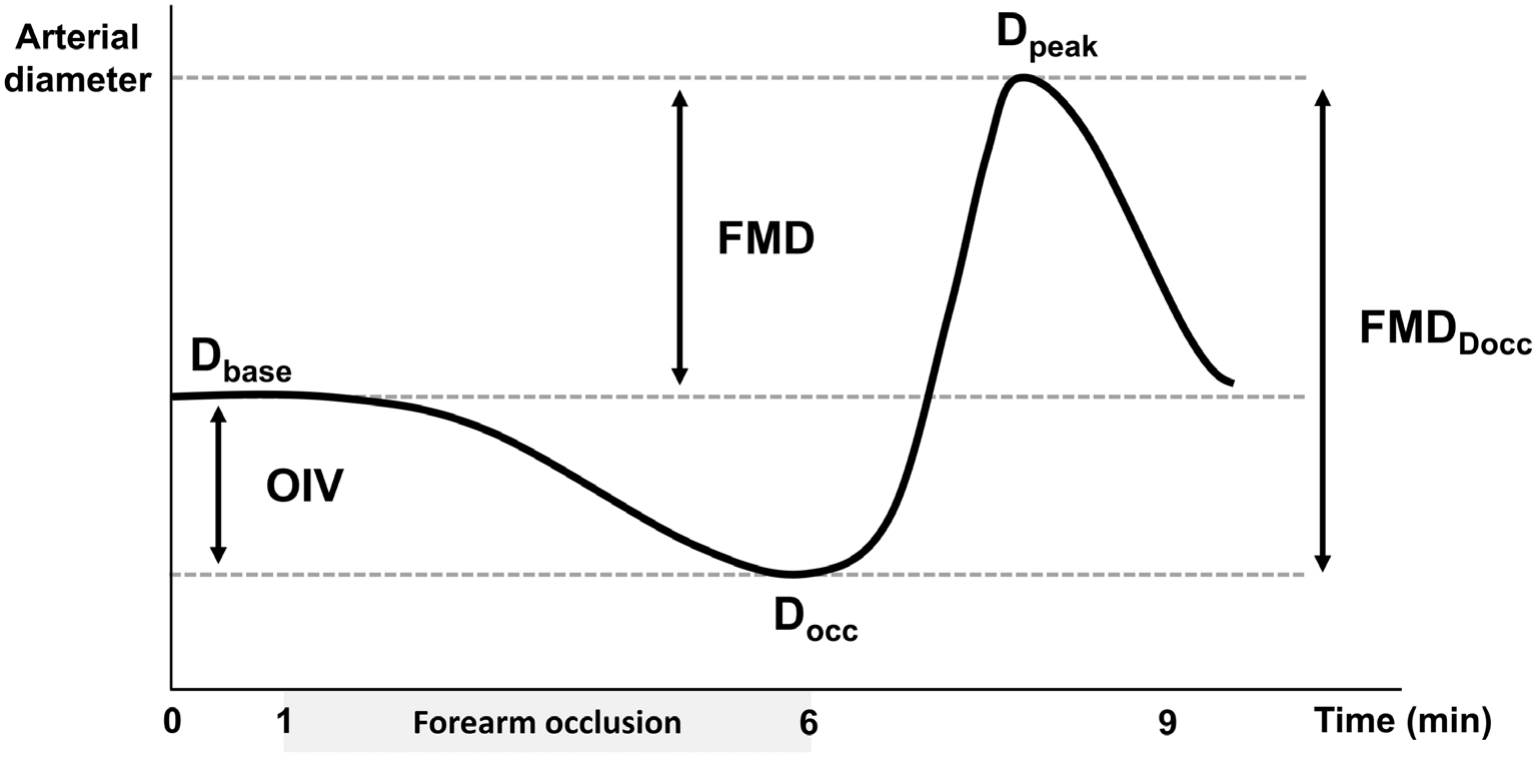
Schematic representation of the change in brachial artery diameter in response to forearm occlusion and the calculation of the associated metrics of vasoactivity. D_base_, baseline diameter; D_occ_, diameter during the final 30 s of occlusion; D_peak_, peak diameter following cuff deflation; FMD, flow‐mediated dilation; FMD_Docc_, flow‐mediated dilation using end‐occlusion diameter instead of baseline diameter; OIV, occlusion‐induced vasoactivity.

Brachial artery diameter is reported at baseline (D_base_), during the final 30 s of occlusion (D_occ_), and the peak diameter following cuff deflation (D_peak_). These variables had a between visit coefficient of variation (CV) at rest of 3.9 ± 3.3%, 4.7 ± 4.0%, and 3.7 ± 3.6% for D_base_, D_occ_, and D_peak_, respectively. The differences between these diameters were calculated as indices of vasoactivity (Figure 1). FMD reflects the difference between D_base_ and D_peak_, while the difference between D_occ_ and D_peak_ is referred to as FMD_Docc_. FMD_Docc_ is included to allow comparisons to FMD as reported/calculated by previous authors post‐heating (Chaseling et al., 2023; Coombs et al., 2021), but to avoid confusion with the traditional calculation of FMD. Finally, as the term LFMC may be misleading in the present context, the difference between D_base_ and D_occ_ is referred to as occlusion‐induced vasoactivity (OIV). These indices were calculated as the absolute change (mm) and as percentage change (%).

### 2.3 Statistical analysis

Interindividual differences in arterial diameter have a known influence on vasoactive responses to occlusion (Atkinson et al., 2013). Accordingly, for percentage change values for indices of vasoactivity (FMD, OIV, and FMD_Docc_), analyses were performed on unadjusted values and adjusted values that were allometrically scaled for arterial diameter and SR_AUC_. To perform the adjusted analysis, the change in logarithmically transformed diameter in response to occlusion was analyzed using a Generalized Estimation Equation (GEE), incorporating arterial diameter and SR_AUC_ as covariates (SR_AUC_ not included in OIV analysis), with the resulting mean differences back transformed to the original units of percentage difference (%). This analysis was performed using SPSS v28.0 (SPSS, Chicago, Illinois) following the steps outlined by Atkinson and Batterham (2013).

Individual estimates of relative changes in arterial diameter adjusted for baseline diameter were then calculated from each participant's residual as derived from observed %—predicted % (as calculated from the GEE) and added to the predicted mean % for each condition and time point at the mean baseline diameter (Lolli et al., 2018). The change from pre to post immersion in vasoactive indices, and diameter variables, along with changes in shear rate from pre to 25 min during immersion (data for this time point reported in Menzies et al. (2025) and are repeated below) were used to perform repeated‐measures correlations using the *rmcorr* package as described by Bakdash and Marusich (2017) to try and explain patterns between variables across all conditions. The remaining statistical analysis was performed using a two‐way (Time × Condition) repeated measures ANOVA using the *anova_test* function from the *Rstatix* package, with effect sizes for main effects presented as partial eta squared ($\eta_{p}^{2}$). Violations of normality were not assessed due to the risks associated with non‐Gaussian models and the robustness of ANOVA in violations of this assumption (Knief & Forstmeier, 2020). Where an interaction effect was identified, post‐hoc analysis between conditions and time points was performed using the *pairwise_t_test* function from the *Rstatix* package, with Bonferroni adjustments for multiple comparisons. Although not the focus of this study, sex differences were analyzed by adding Sex as a between subjects variable into a three‐way (Sex × Time × Condition) ANOVA, the results of which can be found in Appendix S1.

## 3 RESULTS

### 3.1 Thermo‐physiological measures

Rectal and skin temperature increased in all conditions during immersion, as previously reported (Menzies et al., 2025). Rectal temperature increased by 0.9 ± 0.3°C, 0.9 ± 0.3°C, and 0.5 ± 0.1°C for the 40‐Shoulder, 42‐Waist, and 40‐Waist conditions, respectively. This resulted in a lower rectal temperature at the end of immersion in the 40‐Waist condition compared to both the 40‐Shoulder (mean difference: 0.4°C [0.3, 0.5], *p* < 0.001) and 42‐Waist (mean difference: 0.4°C [0.4, 0.5], *p* < 0.001) conditions, which were similar (mean difference: 0.1°C [−0.2, 0.1], *p* = 1.0). During immersion, arm skin temperature increased by 8.1 ± 1.5°C, 3.5 ± 1.1°C, and 2.6 ± 1.0°C, while thigh skin temperature increased by 7.8 ± 1.0°C, 8.6 ± 1.3°C, and 7.8 ± 0.2°C for the 40‐Shoulder, 42‐Waist, and 40‐Waist conditions, respectively.

### 3.2 Brachial artery diameter

**FIGURE 2:**
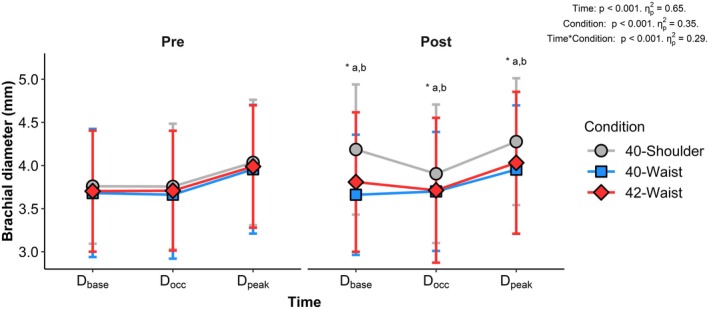
Mean brachial artery diameter pre and post immersion during the FMD assessment for each condition. D_base_, baseline diameter; D_occ_, diameter during the final 30 s of occlusion; D_peak_, peak diameter following cuff deflation. Significance is denoted as follows: ^a^40‐Shoulder versus 42‐Waist (D_base_ *p* < 0.001, D_occ_ *p* = 0.006, D_peak_ *p* < 0.001), ^b^40‐Shoulder versus 40‐Waist (D_base_ *p* < 0.001, D_occ_ *p* = 0.046, D_peak_ *p* < 0.001), ^c^42‐Waist versus 40‐Waist, *versus Pre in the 40‐Shoulder condition at the equivalent timepoint (D_base_ *p* < 0.001, D_occ_ *p* = 0.003, and D_peak_ *p* < 0.001).

Differences in brachial artery diameter between conditions at each time point are shown in Figure 2. D_base_ increased from pre‐immersion in the 40‐Shoulder condition (*p* < 0.001), with no significant change in the 42‐Waist (*p* = 0.15) or 40‐Waist (*p* = 0.78) conditions. As a result, D_base_ was greater post‐immersion in the 40‐Shoulder condition than both the 42‐Waist (mean difference: 0.4 mm [0.2, 0.5], *p* < 0.001) and 40‐Waist (mean difference: 0.5 mm [0.4, 0.7], *p* < 0.001) conditions, which were similar to each other (mean difference: 0.1 mm [0.0, 0.3], *p* = 0.14). D_occ_ was greater post‐immersion compared to pre‐immersion in the 40‐Shoulder condition (*p* = 0.003), with no significant change in the 42‐Waist (*p* = 0.94) or 40‐Waist (*p* = 0.61) conditions. Accordingly, D_occ_ was greater post‐immersion in the 40‐Shoulder condition than both the 42‐Waist (mean difference: 0.2 mm [0.1, 0.3], *p* = 0.006), or 40‐Waist (mean difference: 0.2 mm [0.1, 0.4], *p* = 0.046) conditions, which were similar to each other (mean difference: 0.0 mm [−0.1, 0.2], p = 1.0). D_peak_ increased from pre‐immersion in the 40‐Shoulder condition (*p* < 0.001), but not in the 42‐Waist (*p* = 0.41) or 40‐Waist (*p* = 0.96) conditions. Consequently, D_peak_ was greater post‐immersion in the 40‐Shoulder condition compared with both the 42‐Waist (mean difference: 0.2 mm [0.2, 0.3], *p* < 0.001) and 40‐Waist conditions (mean difference: 0.3 mm [0.2, 0.5], *p* < 0.001), which did not differ from each other (mean difference: 0.1 mm [−0.1, 0.2], *p* = 0.79).

### 3.3 Shear rate and blood flow

Differences in brachial artery shear rate and blood flow between conditions pre‐ and post‐immersion are shown in Table 1. Mean shear rate (pre‐occlusion) was greater post‐immersion compared to pre‐immersion in both the 40‐Shoulder (*p* < 0.001) and 42‐Waist (*p* < 0.001) conditions, but not the 40‐Waist condition (*p* = 0.13). This resulted in greater mean shear rate post‐immersion in the 40‐Shoulder condition compared with both the 42‐Waist (mean difference: 102 1/s [44, 159], *p* = 0.007) and 40‐Waist (mean difference: 227 1/s [176, 278], *p* < 0.001) conditions, while the 42‐Waist condition was greater than the 40‐Waist condition (mean difference: 125 1/s [69, 182], *p* < 0.001). In contrast, compared to pre‐immersion, mean shear rate during occlusion was greater post‐immersion in the 40‐Shoulder condition (*p* < 0.001), but less in the 40‐Waist condition (*p* = 0.01) and did not differ from pre‐immersion in the 42‐Waist condition (*p* = 0.56). As a result, post‐immersion mean shear rate during occlusion differed between all conditions, being greater in the 40‐Shoulder condition than the 42‐Waist (mean difference: 23 1/s [13, 33], *p* < 0.001) and 40‐Waist (mean difference: 35 1/s [26, 44], *p* < 0.001) conditions, with the 42‐Waist condition also being greater than the 40‐Waist condition (mean difference: 12 1/s [6, 19], *p* = 0.003). Mean and antegrade shear rate peaked at 25 min during immersion as previously reported in Menzies et al. (2025) and was different between all conditions at this time point (Mean shear rate: 40‐Shoulder: 455 ± 140 1/s, 42‐Waist: 367 ± 133 1/s, 40‐Waist: 261 ± 110 1/s. Antegrade shear rate: 40‐Shoulder: 457 ± 139 1/s, 42‐Waist: 376 ± 121 1/s, 40‐Waist: 275 ± 102 1/s). SR_AUC_ was greater post‐immersion compared to pre‐immersion in the 40‐Shoulder condition (*p* < 0.001), but was not different to pre‐immersion in either the 42‐Waist (*p* = 0.47), or 40‐Waist (*p* = 0.09) conditions. Post‐immersion SR_AUC_ was smaller in the 40‐Waist condition compared to the 40‐Shoulder (mean difference: 10.3 s^−1^ × 10^3^ [6.2, 14.6], *p* < 0.001) and 42‐Waist (mean difference: 5.5 s^−1^ × 10^3^ [2.0, 8.9], *p* = 0.02) conditions, which were similar (mean difference: 4.9 s^−1^ × 10^3^ [−0.4, 10.3], *p* = 0.26). However, there were no effects of immersion condition on time to peak diameter following occlusion.

**TABLE 1:** Summary of mean blood flow and shear rate responses to forearm occlusion pre and post immersion in each condition.

Variable	Condition	Pre	Post	Statistics
Blood flow (pre occlusion) (mL/min)	40‐Shoulder	28 ± 28	155 ± 96^a,b,^*	Time: *p* < 0.001, $\eta_{p}^{2}$ = 0.58 Condition: *p* < 0.001, $\eta_{p}^{2}$ = 0.53 Time × Condition: *p* < 0.001, $\eta_{p}^{2}$ = 0.54
	42‐Waist	36 ± 35	87 ± 77^a,c,^*	
	40‐Waist	39 ± 42	23 ± 21^b,c,^*	
Blood flow (during occlusion) (mL/min)	40‐Shoulder	13 ± 14	33 ± 19^a,b,^*	Time: *p* < 0.001, $\eta_{p}^{2}$ = 0.42 Condition: *p* < 0.001, $\eta_{p}^{2}$ = 0.46 Time × Condition: *p* < 0.001, $\eta_{p}^{2}$ = 0.52
	42‐Waist	14 ± 11	18 ± 17^a,c^	
	40‐Waist	11 ± 7	7 ± 6^b,c,^*	
Mean shear rate (pre occlusion) (1/s)	40‐Shoulder	43 ± 45	164 ± 163^a,b,^*	Time: *p* < 0.001, $\eta_{p}^{2}$ = 0.50 Condition: *p* < 0.001, $\eta_{p}^{2}$ = 0.53 Time × Condition: *p* < 0.001, $\eta_{p}^{2}$ = 0.57
	42‐Waist	53 ± 54	99 ± 100^a,c,^*	
	40‐Waist	55 ± 57	35 ± 36^b,c^	
Mean shear rate (during occlusion) (1/s)	40‐Shoulder	19 ± 19	46 ± 22^a,b,^*	Time: *p* < 0.001, $\eta_{p}^{2}$ = 0.44 Condition: *p* < 0.001, $\eta_{p}^{2}$ = 0.55 Time × Condition: *p* < 0.001, $\eta_{p}^{2}$ = 0.41
	42‐Waist	21 ± 12	23 ± 18^a,c^	
	40‐Waist	18 ± 11	11 ± 9^b,c,^*	
Antegrade shear rate (pre occlusion) (1/s)	40‐Shoulder	59 ± 35	165 ± 85^a,b,^*	Time: *p* < 0.001, $\eta_{p}^{2}$ = 0.43 Condition: *p* < 0.001, $\eta_{p}^{2}$ = 0.48 Time × Condition: *p* < 0.001, $\eta_{p}^{2}$ = 0.49
	42‐Waist	64 ± 38	107 ± 66^a,c,^*	
	40‐Waist	68 ± 41	51 ± 29^b,c^	
Retrograde shear rate (pre occlusion) (1/s)	40‐Shoulder	‐16 ± 14	0 ± 1^a,b,^*	Time: *p* = 0.003, $\eta_{p}^{2}$ = 0.34 Condition: *p* = 0.002, $\eta_{p}^{2}$ = 0.26 Time × Condition: *p* < 0.001, $\eta_{p}^{2}$ = 0.40
	42‐Waist	−12 ± 10	−8 ± 9^a,c^	
	40‐Waist	−13 ± 10	−17 ± 13^b,c^	
Reactive Hyperaemia SR_AUC_ (s^−1^ × 10^3^)	40‐Shoulder	23.6 ± 8.2	32.2 ± 8.0^b,^*	Time: *p* = 0.07, $\eta_{p}^{2}$ = 0.15 Condition: *p* = 0.008, $\eta_{p}^{2}$ = 0.21 Time × Condition: *p* < 0.001, $\eta_{p}^{2}$ = 0.36
	42‐Waist	25.9 ± 7.6	27.3 ± 8.2^c^	
	40‐Waist	24.6 ± 6.8	21.8 ± 6.3^b,c^	
Time to peak (s)	40‐Shoulder	50 ± 15	62 ± 19	Time: *p* = 0.17, $\eta_{p}^{2}$ = 0.09 Condition: *p* < 0.001, $\eta_{p}^{2}$ = 0.34 Time × Condition: *p* = 0.09, $\eta_{p}^{2}$ = 0.11
	42‐Waist	52 ± 15	56 ± 19	
	40‐Waist	46 ± 15	46 ± 17	

*Note*: Significance is denoted as follows: ^a^40‐Shoulder versus 42‐Waist, ^b^40‐Shoulder versus 40‐Waist, ^c^42‐Waist versus 40‐Waist, and *versus Pre in the same condition.

Abbreviations: SR_AUC_, shear rate area under the curve.

### 3.4 Indices of vasoactivity

**FIGURE 3:**
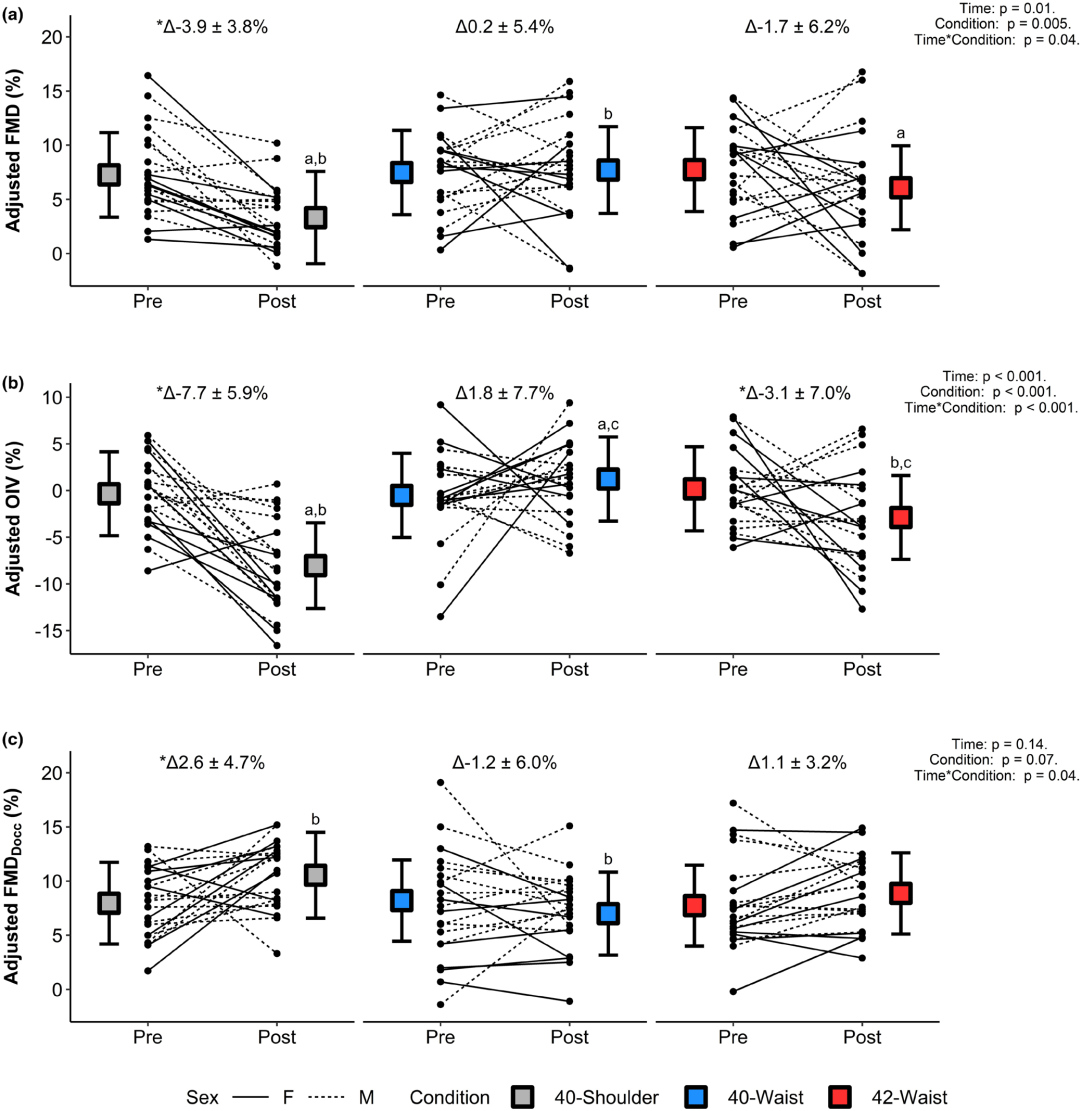
Mean adjusted indices of vasoactivity for each condition pre and post immersion. (a) FMD% (flow‐mediated dilation), (b) OIV% (occlusion‐induced vasoactivity), (c) FMD_Docc_% (flow‐mediated dilation using end‐occlusion diameter instead of baseline). Significance is denoted as follows: ^a^40‐Shoulder versus 42‐Waist (FMD *p* = 0.013, OIV *p* = 0.004), ^b^40‐Shoulder versus 40‐Waist (FMD *p* < 0.001, OIV p < 0.001, FMD_Docc_ *p* = 0.001), ^c^42‐Waist versus 40‐Waist (OIV *p* = 0.032), and *Pre versus Post in the same condition (40‐Shoulder: FMD *p* < 0.001, OIV *p* < 0.001, FMD_Docc_ *p* = 0.029; 42‐Waist: OIV *p* = 0.024).

Absolute (mm) and unadjusted percentage change in all indices of vasoactivity did not show any additional significant condition differences beyond those observed with adjusted percentage changes. Absolute and unadjusted percentage changes can be found in Table 2, while adjusted percentage changes are shown in Figure 3.

**TABLE 2:** Mean absolute and unadjusted percentage changes for indices of vasoactivity for each condition pre and post immersion.

Variable	Condition	Pre	Post	Statistics
Absolute change				
FMD (mm)	40‐Shoulder	0.28 ± 0.15	0.09 ± 0.13^b,^*	Time: *p* = 0.006, $\eta_{p}^{2}$ = 0.31 Condition: *p* = 0.002, $\eta_{p}^{2}$ = 0.25 Time × Condition: *p* = 0.01, $\eta_{p}^{2}$ = 0.20
	42‐Waist	0.29 ± 0.14	0.22 ± 0.21	
	40‐Waist	0.28 ± 0.12	0.29 ± 0.16^b^	
OIV (mm)	40‐Shoulder	0.00 ± 0.15	−0.28 ± 0.17^a,b,^*	Time: *p* = 0.005, $\eta_{p}^{2}$ = 0.31 Condition: *p* < 0.001, $\eta_{p}^{2}$ = 0.39 Time × Condition: p < 0.001, $\eta_{p}^{2}$ = 0.37
	42‐Waist	0.01 ± 0.12	−0.10 ± 0.20^a,c^	
	40‐Waist	−0.02 ± 0.16	0.04 ± 0.15^b,c^	
FMD_Docc_ (mm)	40‐Shoulder	0.28 ± 0.11	0.37 ± 0.15^b,^*	Time: *p* = 0.19, $\eta_{p}^{2}$ = 0.08 Condition: *p* = 0.09, $\eta_{p}^{2}$ = 0.11 Time × Condition: *p* = 0.047, $\eta_{p}^{2}$ = 0.14
	42‐Waist	0.28 ± 0.15	0.32 ± 0.11	
	40‐Waist	0.30 ± 0.19	0.25± 0.10^b^	
Unadjusted percentage change				
FMD (%)	40‐Shoulder	7.4 ± 3.7	2.4 ± 3.1^a,b,^*	Time: *p* = 0.007, $\eta_{p}^{2}$ = 0.30 Condition: *p* < 0.001, $\eta_{p}^{2}$ = 0.32 Time × Condition: *p* = 0.009, $\eta_{p}^{2}$ = 0.20
	42‐Waist	8.1 ± 4.4	6.2 ± 5.1^a^	
	40‐Waist	7.9 ± 4.1	8.2 ± 4.6^b^	
OIV (%)	40‐Shoulder	−0.3 ± 3.8	−7.1 ± 4.4^a,b,^*	Time: p = 0.01, $\eta_{p}^{2}$ = 0.27 Condition: *p* < 0.001, $\eta_{p}^{2}$ = 0.41 Time × Condition: p < 0.001, $\eta_{p}^{2}$ = 0.34
	42‐Waist	−0.2 ± 3.9	−2.7 ± 5.1^a,c^	
	40‐Waist	−0.5 ± 4.6	1.2 ± 4.0^b,c^	
FMD_Docc_ (%)	40‐Shoulder	7.8 ± 3.6	10.4 ± 5.4^b,^*	Time: *p* = 0.29, $\eta_{p}^{2}$ = 0.05 Condition: *p* = 0.17, $\eta_{p}^{2}$ = 0.08 Time × Condition: *p* = 0.03, $\eta_{p}^{2}$ = 0.16
	42‐Waist	7.9 ± 4.5	9.2 ± 4.4	
	40‐Waist	8.7 ± 6.7	6.9 ± 2.7^b^	

*Note*: Significance is denoted as follows: ^a^40‐Shoulder versus 42‐Waist, ^b^40‐Shoulder versus 40‐Waist, ^c^42‐Waist versus 40‐Waist and *versus Pre in the same condition.

Abbreviations: FMD%, flow‐mediated dilation; FMD_Docc_%, flow‐mediated dilation using end‐occlusion diameter instead of baseline; OIV%, occlusion‐induced vasoactivity.

FMD decreased as result of immersion in the 40‐Shoulder condition when measured as absolute change (*p* < 0.001), unadjusted percentage change (p < 0.001), or adjusted percentage change (*p* < 0.001). None of the methods used to calculate FMD showed significant differences between pre‐ and post‐immersion values in either the 42‐Waist (Absolute change: *p* = 0.24. Unadjusted percentage change: *p* = 0.21. Adjusted percentage change: *p* = 0.12), or the 40‐Waist (Absolute change: *p* = 0.71. Unadjusted percentage change: *p* = 0.82. Adjusted percentage change: *p* = 0.83) conditions. Accordingly, adjusted FMD% post‐heating was lower in the 40‐Shoulder condition compared with the 42‐Waist (mean difference: −3% [−5, −1], *p* = 0.01) and 40‐Waist (mean difference: −4% [−6, −2], *p* < 0.01) conditions, which were similar to each other (mean difference: −2% [−4, 0], *p* = 0.14) (Figure 3a).

Similarly, OIV decreased (indicating greater diameter reduction) as result of immersion in the 40‐Shoulder condition when measured as absolute change (*p* < 0.001), unadjusted percentage change (*p* < 0.001), or adjusted percentage change (*p* < 0.001). None of the methods to calculate OIV demonstrated differences between pre‐ and post‐immersion values for the 40‐Waist condition (Absolute change: *p* = 0.33. Unadjusted percentage change: *p* = 0.30. Adjusted percentage change: *p* = 0.19). However, in the 42‐Waist condition, adjusted OIV% was lower post‐immersion than pre‐immersion (indicating greater diameter reduction; *p* = 0.02), whereas OIV expressed as absolute (*p* = 0.07) or unadjusted percentage change (*p* = 0.06) did not differ. Unlike FMD, OIV was different between all conditions post‐immersion across all methods of calculation. Greatest reductions in diameter were observed in the 40‐Shoulder condition (mean difference: Adjusted OIV% vs. 42‐Waist: −5% [−8, −2]. *p* < 0.001. Adjusted OIV% vs. 40‐Waist: −9% [−12, −7]. *p* < 0.001) while the smallest diameter changes were seen in the 40‐Waist condition (mean difference: Adjusted OIV% vs. 42‐Waist: 4% [1, 6]. *p* = 0.003).

All methods of calculating FMD_Docc_ showed that post‐immersion FMD_Docc_ was greater than pre‐immersion values in the 40‐Shoulder condition (Absolute change: *p* = 0.03. Unadjusted percentage change: *p* = 0.03. Adjusted percentage change: *p* = 0.02), but no changes were observed in response to the 42‐Waist (Absolute change: *p* = 0.17. Unadjusted percentage change: *p* = 0.13. Adjusted percentage change: *p* = 0.24), or 40‐Waist (Absolute change: *p* = 0.35. Unadjusted percentage change: *p* = 0.22. Adjusted percentage change: *p* = 0.21) conditions. Differences in post‐immersion adjusted FMD_Docc_% were only seen between the 40‐Shoulder and 40‐Waist conditions (mean difference: 3% [1, 5]. *p* = 0.001) and not between the 40‐Shoulder and 42‐Waist conditions (mean difference: 2% [0, 3]. *p* = 0.10), or the 42‐Waist and 40‐Waist conditions (mean difference: 2% [0, 3]. *p* = 0.06).

### 3.5 Correlations

**FIGURE 4:**
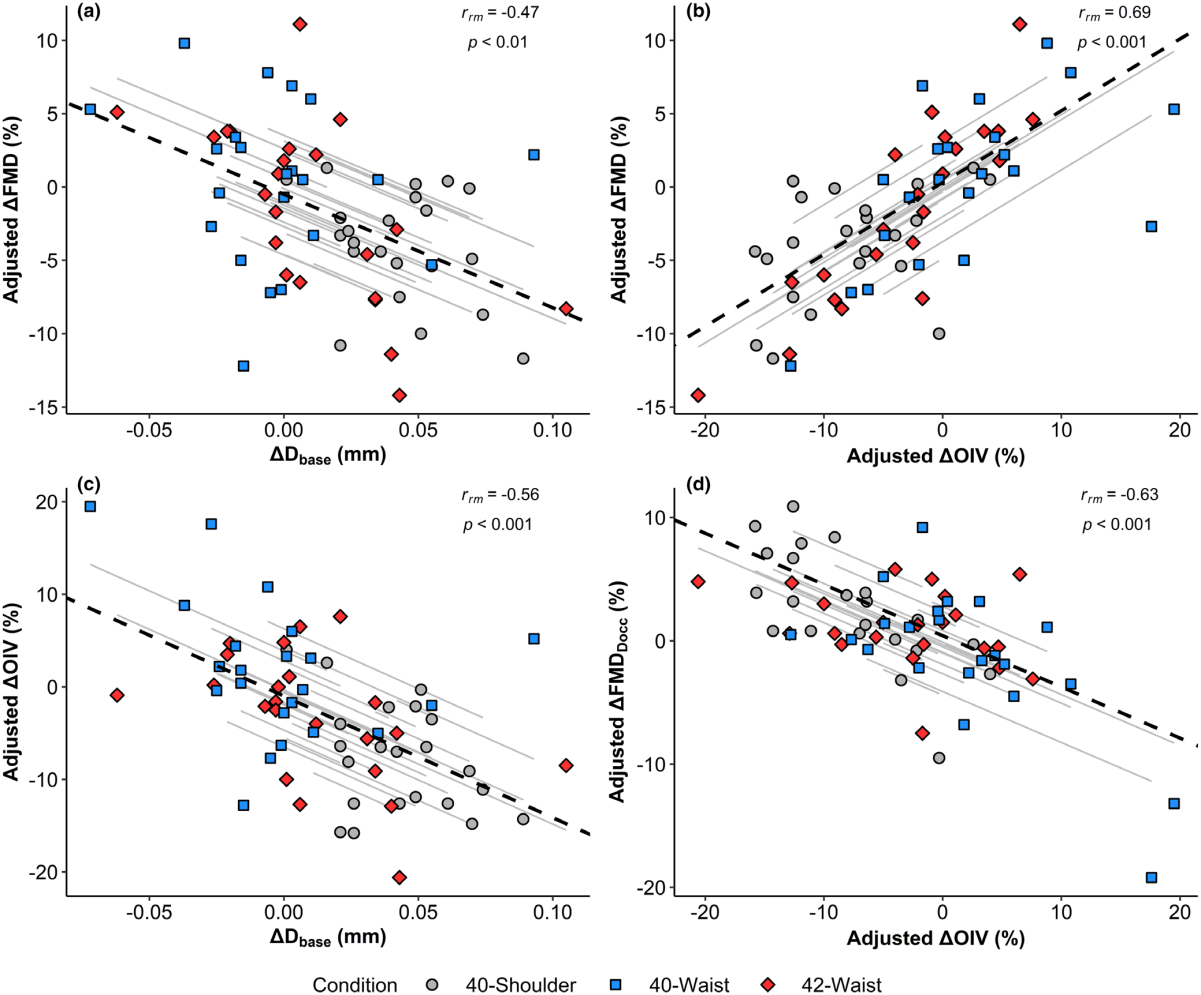
Repeated measures correlations between the changes pre – post immersion in selected indices of vasoactivity. (a) D_base_ versus FMD%, (b) adjusted OIV versus FMD%, (c) D_base_ versus OIV%, and (d) OIV% versus FMD_Docc_%. Smaller gray lines represent the rmcorr fit for each participant, with the dashed black line showing the group average. D_base_, baseline diameter; FMD%, flow‐mediated dilation; FMD_Docc_%, flow‐mediated dilation using end‐occlusion diameter instead of baseline; OIV%, occlusion‐induced vasoactivity.

The pre‐to‐post immersion change in antegrade shear rate displayed a positive correlation with the change in D_base_ (*r*_rm_ = 0.55, *p* < 0.001) and a negative correlation with the change in adjusted FMD% (*r*_rm_ = −0.30, *p* = 0.048). The change in adjusted FMD% was negatively correlated with the change in D_base_ (*r*_rm_ = −0.47, *p* = 0.001) (Figure 4a), and positively correlated with the change in adjusted OIV% (*r*_rm_ = 0.69, *p* < 0.001) (Figure 4b), but showed no relationship with the change in adjusted FMD_Docc_% (*r*_rm_ = 0.11, *p* = 0.46). The change in adjusted OIV% was negatively correlated with the change in D_base_ (*r*_rm_ = −0.56, *p* < 0.001) (Figure 4c), and the change in adjusted FMD_Docc_% (*r*_rm_ = −0.63, *p* < 0.001) (Figure 4d). In contrast, the change in adjusted FMD_Docc_% displayed no relationship with any other variable. A full correlation matrix for key outcomes along with the breakdown by condition is shown in the Appendix S1.

## 4 DISCUSSION

The purpose of the current study was to address the uncertainty surrounding the relative contributions of heat‐induced increases in shear stress and baseline diameter to acute FMD responses. In accordance with our hypothesis, the condition that had the greatest increases in baseline diameter post‐heating (40‐Shoulder) elicited a larger reduction in FMD than the other two conditions. These reductions in FMD% were still present even when allometrically scaled for baseline diameter. Indeed, heat‐induced increases in baseline diameter were negatively correlated with reductions in adjusted FMD% across all conditions (*r*_rm_ = −0.47), demonstrating an effect of increased diameter that is not fully accounted for through allometric scaling. A secondary aim was to examine the acute effects of heating on different indices of vasoactivity to investigate whether these metrics complement or help explain acute changes in FMD. This study shows that, in response to heat‐induced brachial artery vasodilation, the change in diameter during occlusion (OIV) statistically differentiated the effects of water temperature, unlike FMD or absolute changes in baseline diameter. Specifically, higher water temperatures resulted in greater reductions in OIV following waist‐deep immersion. Accordingly, this study provides evidence that OIV is a sensitive complementary metric of vasoactivity in response to acute stimuli, warranting further exploration.

### 4.1 Heat‐induced sustained shear stress, increases in baseline diameter and acute FMD

The present study showed that following 30 min of hot water immersion there were greater reductions in adjusted FMD% in the 40‐Shoulder (∆‐3.9%) than either the 42‐Waist (∆‐1.7%) or 40‐Waist (∆0.2%) conditions. The 40‐Shoulder condition evoked a larger absolute increase in both baseline diameter (D_base_) and peak diameter post‐occlusion (D_peak_) compared to the other two conditions. However, there was a greater effect of heating on baseline diameter (Δ0.43 ± 0.22 mm) than peak diameter post‐occlusion (Δ0.24 ± 0.20 mm) in this condition, which causes the observed reductions in the calculation of adjusted and unadjusted FMD. When combining data from all conditions, there was a significant negative correlation between increases in baseline diameter and decreases in adjusted FMD% (*r*_rm_ = −0.47). In support of this, previous studies have observed larger increases in baseline diameter (~3%–11% vs. ~22%–28%) and subsequently greater reductions in acute FMD (~2%–4% vs. ~6%–8%) in response to a larger heating dose (60 min of heating via a water‐perfused suit) (Alali et al., 2020; Chaseling et al., 2023) than that used in the present study. Moreover, this apparent “dose response” may extend beyond heating protocols; for example, higher exercise intensities also result in larger increases in baseline diameter with subsequent larger decreases in acute FMD (Birk et al., 2013), however, mechanisms such as increased oxidative stress are also suggested to contribute to acute transient vascular dysfunction following high intensity exercise (Dawson et al., 2013). Nevertheless, this study demonstrates a potential confounding effect of sustained heat‐induced increases in baseline diameter on FMD.

The observed increase in baseline diameter in the 40‐Shoulder condition occurs in response to large, sustained elevations in shear stress, with changes in antegrade shear rate showing a significant positive correlation with increases in baseline diameter (*r*_rm_ = 0.55) and a negative correlation with decreases in adjusted FMD% (*r*_rm_ = −0.30). This effect may explain previously observed differences in acute FMD following heating whereby acute increases in antegrade shear rate appear to evoke subsequent increases in FMD (Thijssen et al., 2014; Tinken et al., 2009), unless they are also accompanied by acute increases in baseline diameter (Alali et al., 2020; Birk et al., 2013; Chaseling et al., 2023). It is well documented that arteries with a larger baseline diameter at rest elicit a reduced FMD response, leading to the recommendation of allometric scaling to account for this statistically (Atkinson & Batterham, 2013). However, this study demonstrates a potential confounding effect of sustained heat‐induced increases in baseline diameter on FMD, which persists even after allometric scaling. Indeed, the confounding effect of baseline diameter in the present context does not directly relate to structure per se, rather it is linked to endothelial stimulation and the functional reserve of the vessel, which may explain why allometric scaling does not remove the observed acute effects following heating. Notably, sustained increases in shear stress from passive heating may elicit nitric oxide‐mediated signaling which differs from that triggered by transient increases in shear stress used in standard FMD assessments (Tremblay & Pyke, 2018). Accordingly, the greater increase in baseline diameter in the 40‐Shoulder condition may in itself be interpreted as an acute improvement in endothelial function and emphasizes that the observed reductions in FMD may not represent an impairment in endothelial function as would be traditionally interpreted at rest (Dawson et al., 2013; Thijssen et al., 2009).

### 4.2 Alternate indices of vasoactivity to compliment FMD

The present study showed that vessels with the greatest baseline vasodilation have a larger reserve capacity to fall back on during occlusion, and those with the greatest diameter decrease during occlusion possess the largest reserve to increase following cuff release. This is demonstrated by the relationships between D_base_ and OIV (*r*_rm_ = 0.56) and between OIV and FMD_Docc_ (*r*_rm_ = 0.63). These findings are consistent with previous work showing greater LFMC in larger vessels (Sen et al., 2020) and that longer heating durations elicit greater increases in baseline diameter (Δ0.82 mm), OIV (Δ~14%), and FMD_Docc_ (Δ~11%) than those observed in the present study (Chaseling et al., 2023). Moreover, OIV post‐heating was able to differentiate the effects of immersion depth (i.e., 40‐Shoulder Vs 40‐Waist) and water temperature (i.e., 42‐Waist Vs 40‐Waist), unlike FMD, FMD_Docc_, or absolute changes in baseline diameter demonstrating that it is a sensitive measure of changes in vasoactivity. Accordingly, examining the relative effects of baseline and peak diameter (i.e., FMD) in isolation may overlook key information about the endothelium. By considering the changes in arterial diameter throughout the occlusion protocol, more information may be gained about the vasodilatory reserve and overall vascular environment.

OIV and FMD_Docc_ may provide additional information about the endothelium but require context specific interpretation. For example, consistent with previous studies, this study observed greater reductions in diameter during occlusion (i.e., OIV or LFMC) post‐heating compared to (pre‐heating) resting conditions (Alali et al., 2020; Chaseling et al., 2023). This reduction in diameter observed post‐heating during occlusion is likely due to reduced mechanisms of dilation, rather than inducing constriction per se. This is demonstrated in the 40‐Shoulder condition where both blood flow (Δ5 ± 34 mL/min) and diameter (Δ0.14 ± 0.22 mm) appear to be elevated during occlusion post‐heating compared to pre‐heating resting values. Therefore, despite being mathematically equivalent, OIV and LFMC should be considered separate metrics depending on whether the measurement was performed at rest or following an acute (i.e., heating) stimulus. Similarly, FMD calculated using end‐occlusion rather than baseline diameter (FMD_Docc_) increased post‐heating in the 40‐Shoulder condition in agreement with previous studies (Chaseling et al., 2023; Coombs et al., 2021). The inclusion of FMD_Docc_ as a metric was initially conceived to mitigate the impact of sustained elevations in shear stress on baseline diameter on the “traditional” calculation of FMD (Coombs et al., 2021). However, the change in FMD and FMD_Docc_ showed no relationship with each other (*r*_rm_ = 0.11) in the present study, demonstrating that these are distinct metrics that should not be used interchangeably. SR_AUC_ has been identified as the primary stimulus for FMD at rest (Pyke & Tschakovsky, 2007), however, following heating, this is confounded by the increases in baseline diameter following sustained elevation in shear stress. As FMD_Docc_ measures the immediate changes in diameter following occlusion, this metric may offer some insights into separating the transient effects of SR_AUC_ from sustained elevations in shear stress. More research is required to understand the mechanistic basis of the OIV and FMD_Docc_ responses and to determine their physiological implications; however, they appear to offer distinct information complimentary to FMD regarding the endothelial environment following an acute heating stimulus.

### 4.3 Limitations and future directions

Despite clearly demonstrating the effects of immersion depth and water temperature on acute vasoactive responses and emphasizing the importance of baseline diameter and OIV in the interpretation of this response, the physiological extension of this to repeated exposures and chronic adaptation warrants some caution and further investigation. Additionally, we have previously demonstrated distinct region‐specific arterial shear rate and diameter responses following these protocols (Menzies et al., 2025), and further work should explore the potential interplay between local heated and remote non‐heated regions on the measured acute FMD response. This also means that although brachial artery FMD is reflective of systemic endothelial function at rest (Broxterman et al., 2019), this is unlikely to translate to acute FMD following physiological perturbation (e.g., heating or exercise). Finally, temporal changes in FMD following local and whole‐body heating protocols have previously been reported (Chaseling et al., 2023). While the present study was not designed to investigate these temporal effects, future research should examine whether they differ between the additional indices of vasoactivity reported herein, which could aid our understanding of these metrics and the endothelial response to acute heating.

### 4.4 Summary

This study demonstrated dose‐dependent vasoactive responses to different heating protocols, with 40°C shoulder‐deep immersion producing the greatest reductions in FMD. These effects were driven by larger shear‐stress–induced increases in baseline diameter, which strongly influenced the subsequent FMD response and highlighted the importance of vasodilatory reserve. OIV and FMD_Docc_ were identified as distinct but complementary metrics of vasoactivity to FMD that warrant inclusion in future studies of endothelial responses to acute physiological stimuli, with OIV proving the only metric of vasoactivity that distinguished between all heating conditions in the present study.

## AUTHOR CONTRIBUTIONS

Campbell Menzies, Tom Cullen, Neil Clarke, Christopher Pugh, and Doug Thake were responsible for the conception and design of the study. Campbell Menzies, Charles Steward, and Tom Cullen were responsible for data acquisition, while all authors assisted in the interpretation of the data. All authors contributed to drafting or revision of the written work, approved the final version, and agree to be accountable for all aspects of the work in ensuring that questions related to the accuracy or integrity of any part of the work are appropriately investigated and resolved. All persons designated as authors qualify for authorship, and all those who qualify for authorship are listed.

## FUNDING INFORMATION

This work received no external funding.

## CONFLICT OF INTEREST STATEMENT

The authors report no conflict of interest.

## ETHICS STATEMENT

This study was approved by the Coventry University Ethics committee (P146084) and conformed to the Declaration of Helsinki, except for prior registration in a database.

## PATIENT CONSENT STATEMENT

Written informed consent was obtained from all participants prior to taking part in the study.

## Supporting information


Appendix S1.

## Data Availability

Data available in the Appendix S1.
