# Supplementary material for: Acute measurement of flow‐mediated dilation following passive heating in adults: The confounding role of altered shear stress and baseline vasodilation
Source: Physiol Rep. 2026 Feb 16;14(4):e70723. doi: 10.14814/phy2.70723 (PMC12910132; doi:10.14814/phy2.70723)
Supplement: Supplementary file 1 — Appendix S1. [file PHY2-14-e70723-s001.docx]

# Supplementary material

## Correlation matrix for vasoactivity outcomes

1. Rectal temperature

|  | r_rm_ | p | CI | |
| --- | --- | --- | --- | --- |
| Dbase | 0.450 | 0.002 | 0.180 | 0.657 |
| OIV | -0.377 | 0.011 | -0.094 | -0.604 |
| FMD | -0.250 | 0.098 | -0.506 | 0.047 |
| FMDDocc | 0.220 | 0.146 | -0.078 | 0.483 |
| SR | 0.368 | 0.013 | 0.083 | 0.597 |
| Antegrade SR | 0.467 | 0.001 | 0.201 | 0.669 |
| Retrograde SR | 0.277 | 0.065 | -0.018 | 0.528 |
| SRAUC | 0.371 | 0.012 | 0.087 | 0.599 |
| SR_occ | -0.458 | 0.002 | -0.663 | -0.191 |

1. D_base_

|  | r_rm_ | p | CI | |
| --- | --- | --- | --- | --- |
| FMD | -0.469 | 0.001 | -0.670 | -0.203 |
| OIV | -0.559 | 0.000 | -0.318 | -0.732 |
| FMDDocc | 0.253 | 0.094 | -0.044 | 0.509 |
| SR | 0.441 | 0.002 | 0.169 | 0.650 |
| Antegrade_SR | 0.550 | 0.000 | 0.306 | 0.727 |
| Retrograde_SR | 0.194 | 0.201 | -0.105 | 0.462 |

1. OIV

|  | r_rm_ | p | CI | |
| --- | --- | --- | --- | --- |
| FMD | 0.690 | 0.000 | 0.818 | 0.497 |
| FMDDocc | -0.634 | 0.000 | -0.419 | -0.782 |
| SR | -0.328 | 0.028 | -0.567 | -0.039 |
| Antegrade_SR | -0.448 | 0.002 | -0.178 | -0.656 |
| Retrograde_SR | -0.254 | 0.092 | 0.043 | -0.510 |
| SRAUC | -0.431 | 0.003 | -0.157 | -0.643 |
| SR_occ | 0.442 | 0.002 | 0.651 | 0.170 |

1. FMD

|  | r_rm_ | p | CI | |
| --- | --- | --- | --- | --- |
| FMDDocc | 0.114 | 0.458 | -0.186 | 0.394 |
| SR | -0.216 | 0.154 | -0.479 | 0.083 |
| Antegrade_SR | -0.296 | 0.048 | -0.543 | -0.003 |
| Retrograde_SR | -0.147 | 0.334 | -0.423 | 0.153 |
| SRAUC | -0.178 | 0.242 | -0.448 | 0.122 |
| SR_occ | 0.295 | 0.050 | 0.001 | 0.541 |

1. FMD_Docc_

|  | r_rm_ | p | CI | |
| --- | --- | --- | --- | --- |
| SR | 0.190 | 0.212 | -0.110 | 0.458 |
| Antegrade_SR | 0.265 | 0.078 | -0.031 | 0.518 |
| Retrograde_SR | 0.149 | 0.330 | -0.152 | 0.424 |
| SRAUC | 0.290 | 0.053 | -0.004 | 0.538 |
| SR_occ | -0.261 | 0.083 | -0.515 | 0.035 |

## Correlations by condition – significant correlations shown in bold

1. 40-Shoulder

| r values | FMD  change | OIV  change | FMDDocc  change | SRAUC  change | SR  change | Antegrade_SR  change | Retrograde_SR  change | Mean_SR_occ  change | Trec  change |
| --- | --- | --- | --- | --- | --- | --- | --- | --- | --- |
| Dbase  change | -0.34 | -0.41 | 0.13 | 0.40 | -0.31 | -0.24 | 0.24 | 0.27 | 0.21 |
| FMD  change |  | **0.43** | 0.32 | -0.11 | -0.01 | 0.09 | -0.09 | -0.10 | 0.00 |
| OIV  change |  |  | **-0.71** | **-0.62** | -0.08 | -0.10 | -0.07 | 0.09 | 0.12 |
| FMDDocc  change |  |  |  | **0.49** | 0.10 | 0.22 | -0.03 | -0.22 | -0.15 |
| SRAUC  change |  |  |  |  | -0.07 | -0.16 | 0.15 | 0.17 | 0.16 |
| SR  change |  |  |  |  |  | **0.92** | 0.27 | **-0.89** | 0.03 |
| Antegrade_SR  change |  |  |  |  |  |  | 0.08 | **-0.99** | -0.05 |
| Retrograde_SR  change |  |  |  |  |  |  |  | 0.03 | 0.16 |
| Mean_SR_occ  change |  |  |  |  |  |  |  |  | 0.07 |

| p values | FMD  change | OIV  change | FMDDocc  change | SRAUC  change | SR  change | Antegrade_SR  change | Retrograde_SR  change | Mean_SR_occ  change | Trec  change |
| --- | --- | --- | --- | --- | --- | --- | --- | --- | --- |
| Dbase  change | 0.1222 | 0.0600 | 0.5530 | 0.0668 | 0.1629 | 0.2732 | 0.2924 | 0.2247 | 0.3435 |
| FMD  change |  | **0.0456** | 0.1469 | 0.6315 | 0.9796 | 0.6898 | 0.6861 | 0.6572 | 0.9830 |
| OIV  change |  |  | **0.0002** | **0.0022** | 0.7075 | 0.6559 | 0.7663 | 0.6779 | 0.6003 |
| FMDDocc  change |  |  |  | **0.0221** | 0.6471 | 0.3342 | 0.8839 | 0.3246 | 0.4923 |
| SRAUC  change |  |  |  |  | 0.7445 | 0.4859 | 0.5190 | 0.4430 | 0.4873 |
| SR  change |  |  |  |  |  | **0.0000** | 0.2298 | **0.0000** | 0.8842 |
| Antegrade_SR  change |  |  |  |  |  |  | 0.7396 | **0.0000** | 0.8276 |
| Retrograde_SR  change |  |  |  |  |  |  |  | 0.8915 | 0.4662 |
| Mean_SR_occ  change |  |  |  |  |  |  |  |  | 0.7681 |

1. 42-Waist

| r values | FMD  change | OIV  change | FMDDocc  change | SRAUC  change | SR  change | Antegrade_SR  change | Retrograde_SR  change | Mean_SR_occ  change | Trec  change |
| --- | --- | --- | --- | --- | --- | --- | --- | --- | --- |
| Dbase  change | **-0.63** | **-0.48** | -0.24 | 0.03 | 0.09 | -0.05 | -0.03 | 0.05 | 0.38 |
| FMD  change |  | **0.89** | 0.16 | -0.27 | -0.29 | -0.25 | 0.14 | 0.29 | -0.24 |
| OIV  change |  |  | -0.31 | -0.17 | -0.37 | -0.36 | 0.09 | 0.39 | -0.19 |
| FMDDocc  change |  |  |  | -0.32 | 0.18 | 0.18 | 0.10 | -0.18 | -0.10 |
| SRAUC  change |  |  |  |  | 0.22 | 0.21 | -0.33 | -0.28 | 0.12 |
| SR  change |  |  |  |  |  | 0.20 | -0.13 | -0.24 | -0.05 |
| Antegrade_SR  change |  |  |  |  |  |  | **0.43** | **-0.99** | 0.2 |
| Retrograde_SR  change |  |  |  |  |  |  |  | -0.28 | 0.36 |
| Mean_SR_occ  change |  |  |  |  |  |  |  |  | -0.14 |

| p values | FMD  change | OIV  change | FMDDocc  change | SRAUC  change | SR  change | Antegrade_SR  change | Retrograde_SR  change | Mean_SR_occ  change | Trec  change |
| --- | --- | --- | --- | --- | --- | --- | --- | --- | --- |
| Dbase  change | **0.0016** | **0.0248** | 0.2785 | 0.8814 | 0.6841 | 0.8149 | 0.8893 | 0.8233 | 0.0820 |
| FMD  change |  | **0.0000** | 0.4851 | 0.2302 | 0.1870 | 0.2672 | 0.5413 | 0.1945 | 0.2895 |
| OIV  change |  |  | 0.1583 | 0.4560 | 0.0878 | 0.1034 | 0.7054 | 0.0694 | 0.3935 |
| FMDDocc  change |  |  |  | 0.1490 | 0.4201 | 0.4157 | 0.6593 | 0.4321 | 0.6420 |
| SRAUC  change |  |  |  |  | 0.3298 | 0.3601 | 0.1323 | 0.2122 | 0.5880 |
| SR  change |  |  |  |  |  | 0.3646 | 0.5747 | 0.2852 | 0.8346 |
| Antegrade_SR  change |  |  |  |  |  |  | **0.0467** | **0.0000** | 0.3820 |
| Retrograde_SR  change |  |  |  |  |  |  |  | 0.2110 | 0.0951 |
| Mean_SR_occ  change |  |  |  |  |  |  |  |  | 0.5239 |

1. 40-Waist

| r values | FMD  change | OIV  change | FMDDocc  change | SRAUC  change | SR  change | Antegrade_SR  change | Retrograde_SR  change | Mean_SR_occ  change | Trec  change |
| --- | --- | --- | --- | --- | --- | --- | --- | --- | --- |
| Dbase  change | -0.15 | -0.36 | 0.34 | 0.09 | 0.08 | 0.14 | -0.33 | -0.20 | **0.44** |
| FMD  change |  | **0.60** | 0.14 | 0.26 | 0.21 | 0.23 | -0.04 | -0.24 | -0.05 |
| OIV  change |  |  | **-0.70** | 0.07 | 0.21 | 0.23 | -0.03 | -0.24 | **-0.44** |
| FMDDocc  change |  |  |  | 0.07 | -0.06 | -0.07 | -0.01 | 0.06 | **0.48** |
| SRAUC  change |  |  |  |  | 0.00 | 0.01 | -0.07 | -0.03 | 0.26 |
| SR  change |  |  |  |  |  | **0.99** | 0.33 | **-0.94** | -0.04 |
| Antegrade_SR  change |  |  |  |  |  |  | 0.17 | **-0.98** | -0.04 |
| Retrograde_SR  change |  |  |  |  |  |  |  | 0.01 | 0.00 |
| Mean_SR_occ  change |  |  |  |  |  |  |  |  | 0.04 |

| p values | FMD  change | OIV  change | FMDDocc  change | SRAUC  change | SR  change | Antegrade_SR  change | Retrograde_SR  change | Mean_SR_occ  change | Trec  change |
| --- | --- | --- | --- | --- | --- | --- | --- | --- | --- |
| Dbase  change | 0.4947 | 0.0994 | 0.1162 | 0.7028 | 0.7259 | 0.5318 | 0.1301 | 0.3671 | **0.0418** |
| FMD  change |  | **0.0031** | 0.5211 | 0.2453 | 0.3452 | 0.3069 | 0.8431 | 0.2828 | 0.8085 |
| OIV  change |  |  | **0.0003** | 0.7488 | 0.3407 | 0.3062 | 0.8819 | 0.2863 | **0.0414** |
| FMDDocc  change |  |  |  | 0.7522 | 0.7767 | 0.7725 | 0.9619 | 0.7757 | **0.0243** |
| SRAUC  change |  |  |  |  | 0.9959 | 0.9519 | 0.7612 | 0.9080 | 0.2396 |
| SR  change |  |  |  |  |  | **0.0000** | 0.1333 | **0.0000** | 0.8572 |
| Antegrade_SR  change |  |  |  |  |  |  | 0.4498 | **0.0000** | 0.8509 |
| Retrograde_SR  change |  |  |  |  |  |  |  | 0.9815 | 0.9917 |
| Mean_SR_occ  change |  |  |  |  |  |  |  |  | 0.8473 |

## Sex differences

1. Rectal temperature

| Condition | Sex | 0 | 5 | 10 | 15 | 20 | 25 | 30 | 40 |
| --- | --- | --- | --- | --- | --- | --- | --- | --- | --- |
| 40-Shoulder | F | 37.0 (0.3) | 37.0 (0.3) | 37.0 (0.3) | 37.2 (0.3) | 37.4 (0.3) | 37.8 (0.3) | 37.9 (0.3) | 37.8 (0.3) |
| 40-Shoulder | M | 36.8 (0.3) | 36.9 (0.3) | 37.0 (0.3) | 37.2 (0.3) | 37.3 (0.3) | 37.6 (0.3) | 37.8 (0.3) | 37.6 (0.3) |
| 40-Waist | F | 37.0 (0.3) | 37.1 (0.3) | 37.2 (0.3) | 37.2 (0.3) | 37.3 (0.3) | 37.4 (0.3) | 37.6 (0.3) | 37.5 (0.3) |
| 40-Waist | M | 36.8 (0.2) | 36.9 (0.2) | 36.9 (0.2) | 37.0 (0.2) | 37.1 (0.2) | 37.2 (0.2) | 37.3 (0.2) | 37.3 (0.2) |
| 42-Waist | F | 37.1 (0.2) | 37.2 (0.2) | 37.3 (0.2) | 37.5 (0.3) | 37.7 (0.3) | 37.9 (0.3) | 38.1 (0.3) | 37.7 (0.2) |
| 42-Waist | M | 36.8 (0.1) | 36.9 (0.1) | 37.1 (0.1) | 37.3 (0.2) | 37.4 (0.2) | 37.6 (0.2) | 37.7 (0.2) | 37.6 (0.2) |


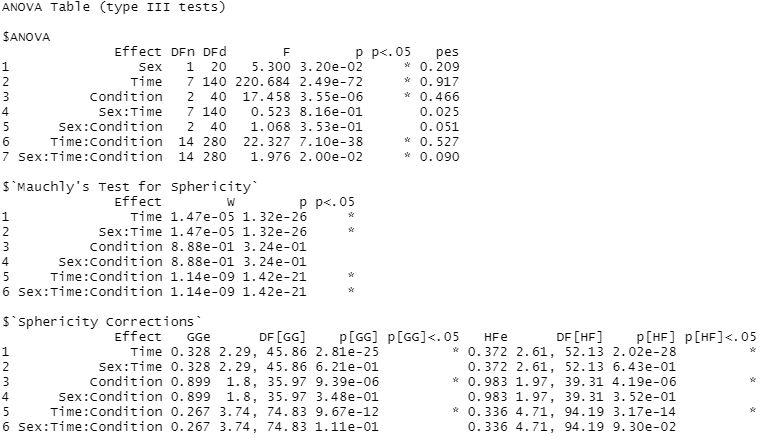


1. FMD absolute

| Condition | Sex | Pre | Post |
| --- | --- | --- | --- |
| 40-Shoulder | F | 0.02 (0.01) | 0.01 (0.01) |
| 40-Shoulder | M | 0.03 (0.01) | 0.01 (0.02) |
| 40-Waist | F | 0.03 (0.01) | 0.03 (0.01) |
| 40-Waist | M | 0.03 (0.01) | 0.03 (0.02) |
| 42-Waist | F | 0.03 (0.02) | 0.02 (0.01) |
| 42-Waist | M | 0.03 (0.01) | 0.02 (0.02) |


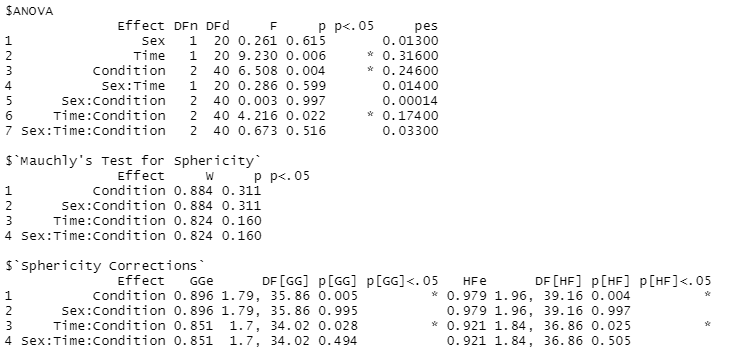


1. FMD relative (unadjusted)

| Condition | Sex | Pre | Post |
| --- | --- | --- | --- |
| 40-Shoulder | F | 6.35 (4.32) | 2.34 (1.9) |
| 40-Shoulder | M | 8.00 (3.58) | 4.05 (3.22) |
| 40-Waist | F | 7.69 (4.2) | 6.59 (4.49) |
| 40-Waist | M | 7.43 (3.34) | 8.64 (4.58) |
| 42-Waist | F | 7.75 (4.99) | 4.75 (4.15) |
| 42-Waist | M | 7.86 (3.32) | 7.17 (5.3) |


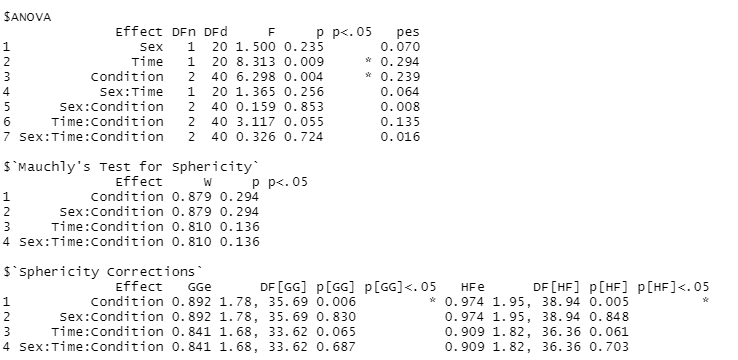


1. FMD_Docc_ absolute

| Condition | Sex | Pre | Post |
| --- | --- | --- | --- |
| 40-Shoulder | F | 0.03 (0.01) | 0.04 (0.01) |
| 40-Shoulder | M | 0.03 (0.01) | 0.03 (0.02) |
| 40-Waist | F | 0.03 (0.02) | 0.02 (0.01) |
| 40-Waist | M | 0.03 (0.02) | 0.03 (0.01) |
| 42-Waist | F | 0.03 (0.01) | 0.04 (0.01) |
| 42-Waist | M | 0.03 (0.02) | 0.03 (0.01) |


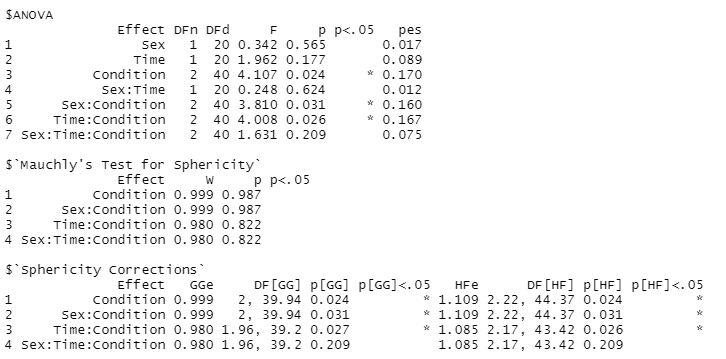


1. FMD_Docc_ relative (unadjusted)

| Condition | Sex | Pre | Post |
| --- | --- | --- | --- |
| 40-Shoulder | F | 7.83 (3.58) | 11.53 (2.68) |
| 40-Shoulder | M | 8.13 (3.07) | 9.93 (3.32) |
| 40-Waist | F | 7.68 (6.81) | 4.36 (3.14) |
| 40-Waist | M | 8.79 (5.14) | 8.92 (2.55) |
| 42-Waist | F | 6.41 (3.99) | 8.72 (4.54) |
| 42-Waist | M | 8.78 (4.00) | 9.05 (2.27) |


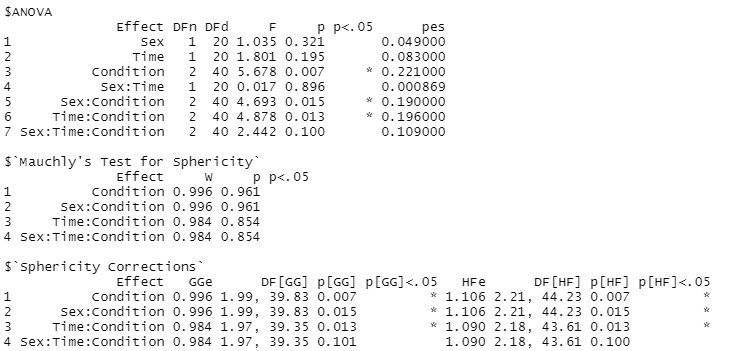


1. OIV absolute

| Condition | Sex | Pre | Post |
| --- | --- | --- | --- |
| 40-Shoulder | F | 0.007 (0.014) | 0.034 (0.012) |
| 40-Shoulder | M | -0.004 (0.014) | 0.024 (0.019) |
| 40-Waist | F | 0.001 (0.018) | -0.005 (0.01) |
| 40-Waist | M | 0.003 (0.014) | -0.003 (0.018) |
| 42-Waist | F | -0.003 (0.014) | 0.016 (0.015) |
| 42-Waist | M | 0.001 (0.011) | 0.005 (0.023) |


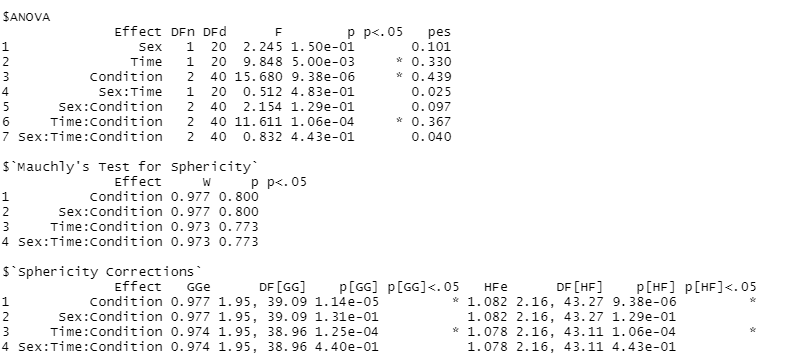


1. OIV relative (unadjusted)

| Condition | Sex | Pre | Post |
| --- | --- | --- | --- |
| 40-Shoulder | F | -1.94 (4.21) | -9.93 (3.1) |
| 40-Shoulder | M | 0.86 (3.19) | -5.13 (4.22) |
| 40-Waist | F | -0.57 (6.07) | 1.74 (3.37) |
| 40-Waist | M | -0.48 (3.48) | 0.86 (4.55) |
| 42-Waist | F | 0.69 (5.01) | -5.12 (4.37) |
| 42-Waist | M | -0.12 (3.1) | -0.97 (5) |


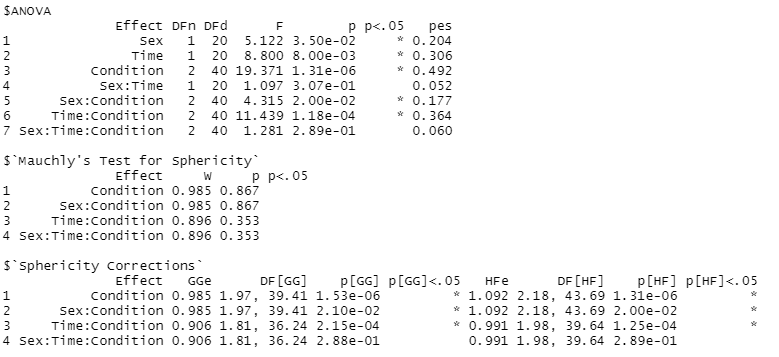


1. Brachial diameter during and following occlusion

| Condition | Sex | Dbase_Pre | Docc_Pre | Dpeak_Pre | Dbase_Post | Docc_Post | Dpeak_Post |
| --- | --- | --- | --- | --- | --- | --- | --- |
| 40-Shoulder | F | 0.32 (0.05) | 0.31 (0.05) | 0.34 (0.05) | 0.35 (0.06) | 0.31 (0.05) | 0.36 (0.05) |
| 40-Shoulder | M | 0.42 (0.04) | 0.42 (0.05) | 0.45 (0.05) | 0.47 (0.04) | 0.44 (0.05) | 0.48 (0.04) |
| 40-Waist | F | 0.30 (0.06) | 0.30 (0.06) | 0.33 (0.06) | 0.30 (0.04) | 0.31 (0.04) | 0.33 (0.04) |
| 40-Waist | M | 0.42 (0.04) | 0.41 (0.03) | 0.44 (0.04) | 0.41 (0.05) | 0.41 (0.05) | 0.44 (0.05) |
| 42-Waist | F | 0.3. (0.05) | 0.31 (0.06) | 0.33 (0.05) | 0.31 (0.05) | 0.29 (0.05) | 0.33 (0.05) |
| 42-Waist | M | 0.42 (0.04) | 0.41 (0.04) | 0.44 (0.04) | 0.43 (0.05) | 0.43 (0.05) | 0.46 (0.05) |


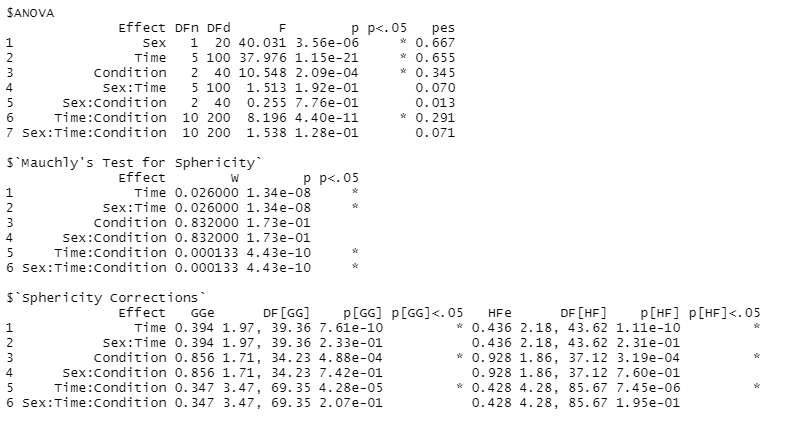


1. SR_AUC_

| Condition | Sex | Pre | Post |
| --- | --- | --- | --- |
| 40-Shoulder | F | 23306 (7746) | 34074 (10714) |
| 40-Shoulder | M | 23858 (8819) | 30940 (5691) |
| 40-Waist | F | 24700 (6652) | 23647 (5632) |
| 40-Waist | M | 24499 (7118) | 20598 (6648) |
| 42-Waist | F | 25618 (5106) | 29928 (7059) |
| 42-Waist | M | 26127 (9099) | 25497 (8687) |


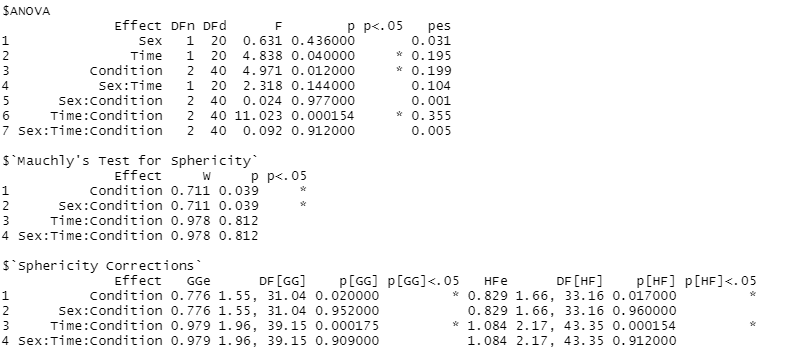


1. Time to peak

| Condition | Sex | Pre | Post |
| --- | --- | --- | --- |
| 40-Shoulder | F | 45 (14) | 52 (15) |
| 40-Shoulder | M | 53 (16) | 68 (19) |
| 40-Waist | F | 39 (10) | 43 (13) |
| 40-Waist | M | 51 (14) | 48 (20) |
| 42-Waist | F | 46 (14) | 46 (10) |
| 42-Waist | M | 57 (14) | 63 (21) |


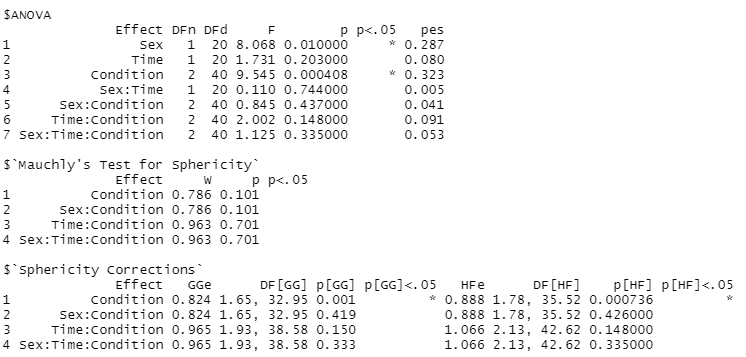


## Data

Rectal temp

|  | 40-Shoulder | 40-Shoulder | 42-Waist | 42-Waist | 40-Waist | 40-Waist |
| --- | --- | --- | --- | --- | --- | --- |
|  | Pre | Post | Pre | Post | Pre | Post |
| 1 | 36.9 | 37.9 | 36.8 | 38.2 | 37.0 | 37.5 |
| 2 | 36.8 | 37.7 | 37.0 | 37.7 | 36.8 | 37.4 |
| 3 | 36.6 | 37.6 | 36.9 | 37.6 | 36.8 | 37.3 |
| 4 | 37.0 | 38.2 | 37.1 | 38.0 | 36.9 | 37.5 |
| 5 | 36.8 | 37.6 | 36.9 | 37.6 | 36.7 | 37.2 |
| 6 | 37.2 | 37.7 | 37.2 | 37.8 | 37.0 | 37.5 |
| 7 | 37.4 | 37.8 | 37.4 | 37.9 | 37.1 | 37.4 |
| 8 | 36.8 | 38.1 | 36.9 | 37.7 | 36.6 | 37.1 |
| 9 | 37.0 | 37.8 | 36.8 | 37.5 | 37.1 | 37.6 |
| 10 | 37.0 | 37.8 | 37.1 | 37.8 | 37.2 | 37.6 |
| 11 | 36.8 | 37.6 | 37.0 | 37.6 | 37.0 | 37.4 |
| 12 | 36.5 | 37.5 | 36.9 | 37.7 | 36.4 | 36.9 |
| 13 | 37.2 | 38.2 | 37.1 | 38.4 | 37.3 | 38.0 |
| 14 | 37.2 | 38.1 | 37.0 | 38.3 | 37.1 | 37.7 |
| 15 | 36.9 | 38.0 | 37.3 | 38.5 | 37.4 | 37.8 |
| 16 | 36.9 | 37.5 | 36.8 | 38.0 | 36.8 | 37.4 |
| 17 | 37.7 | 38.3 | 36.9 | 37.8 | 37.0 | 37.4 |
| 18 | 36.6 | 37.6 | 36.7 | 37.8 | 36.7 | 37.3 |
| 19 | 36.8 | 38.0 | 36.8 | 37.9 | 36.7 | 37.4 |
| 20 | 36.8 | 37.9 | 36.8 | 37.9 | 36.7 | 37.3 |
| 21 | 36.8 | 38.3 | 37.1 | 38.2 | 37.3 | 37.9 |
| 22 | 36.6 | 37.3 | 36.7 | 37.5 | 36.6 | 37.0 |

Mean shear rate

| Condition | 40-Shoulder | 40-Shoulder | 42-Waist | 42-Waist | 40-Waist | 40-Waist |
| --- | --- | --- | --- | --- | --- | --- |
| Time | Pre | Post | Pre | Post | Pre | Post |
| 1 | 8 | 191 | 31 | 13 | 13 | 83 |
| 2 | 17 | 114 | 46 | 13 | 31 | 65 |
| 3 | 16 | 141 | 26 | 32 | 38 | 147 |
| 4 | 24 | 126 | 50 | 108 | 18 | 50 |
| 5 | 40 | 148 | 52 | 10 | 97 | 68 |
| 6 | 15 | 363 | 22 | 71 | 70 | 67 |
| 7 | 75 | 114 | 30 | 16 | 35 | 39 |
| 8 | 28 | 176 | 20 | 25 | 12 | 37 |
| 9 | 14 | 82 | 27 | 4 | 34 | 5 |
| 10 | 64 | 137 | 73 | 16 | 32 | 97 |
| 11 | 69 | 97 | 32 | 15 | 141 | 145 |
| 12 | 25 | 194 | 28 | 9 | 30 | 115 |
| 13 | 49 | 190 | 62 | 28 | 82 | 123 |
| 14 | 28 | 173 | 23 | 32 | 55 | 108 |
| 15 | 65 | 120 | 58 | 28 | 82 | 12 |
| 16 | 52 | 22 | 54 | 12 | 32 | 42 |
| 17 | 136 | 119 | 101 | 56 | 114 | 108 |
| 18 | 17 | 233 | 20 | 35 | 36 | 64 |
| 19 | 52 | 147 | 178 | 56 | 45 | 161 |
| 20 | 19 | 73 | 70 | 85 | 33 | 122 |
| 21 | 41 | 345 | 67 | 42 | 21 | 308 |
| 22 | 93 | 310 | 150 | 53 | 105 | 220 |

Antegrade shear rate

| Condition | 40-Shoulder | 40-Shoulder | 42-Waist | 42-Waist | 40-Waist | 40-Waist |
| --- | --- | --- | --- | --- | --- | --- |
| Time | Pre | Post | Pre | Post | Pre | Post |
| 1 | 14 | 191 | 22 | 87 | 36 | 15 |
| 2 | 34 | 114 | 68 | 80 | 80 | 34 |
| 3 | 28 | 141 | 54 | 149 | 57 | 43 |
| 4 | 30 | 126 | 20 | 56 | 58 | 120 |
| 5 | 80 | 148 | 104 | 80 | 84 | 47 |
| 6 | 19 | 363 | 84 | 80 | 37 | 112 |
| 7 | 86 | 115 | 38 | 70 | 43 | 35 |
| 8 | 45 | 176 | 20 | 44 | 30 | 37 |
| 9 | 18 | 82 | 38 | 13 | 29 | 6 |
| 10 | 68 | 137 | 42 | 112 | 87 | 30 |
| 11 | 123 | 97 | 152 | 162 | 50 | 25 |
| 12 | 32 | 194 | 36 | 117 | 36 | 24 |
| 13 | 69 | 190 | 106 | 123 | 85 | 68 |
| 14 | 60 | 173 | 74 | 108 | 27 | 51 |
| 15 | 87 | 120 | 110 | 31 | 72 | 74 |
| 16 | 69 | 25 | 61 | 63 | 80 | 32 |
| 17 | 138 | 119 | 123 | 108 | 101 | 59 |
| 18 | 19 | 233 | 36 | 64 | 21 | 35 |
| 19 | 61 | 147 | 48 | 161 | 188 | 64 |
| 20 | 33 | 73 | 37 | 125 | 72 | 85 |
| 21 | 75 | 346 | 29 | 312 | 79 | 61 |
| 22 | 100 | 310 | 107 | 220 | 154 | 66 |

Retrograde shear rate

| Condition | 40-Shoulder | 40-Shoulder | 42-Waist | 42-Waist | 40-Waist | 40-Waist |
| --- | --- | --- | --- | --- | --- | --- |
| Time | Pre | Post | Pre | Post | Pre | Post |
| 1 | -6 | 0 | -9 | -4 | -4 | -2 |
| 2 | -18 | 0 | -36 | -15 | -34 | -21 |
| 3 | -12 | 0 | -16 | -2 | -30 | -11 |
| 4 | -5 | 0 | -3 | -6 | -8 | -11 |
| 5 | -39 | 0 | -7 | -12 | -32 | -37 |
| 6 | -3 | 0 | -13 | -13 | -15 | -41 |
| 7 | -12 | -1 | -3 | -31 | -13 | -20 |
| 8 | -17 | 0 | -8 | -7 | -9 | -12 |
| 9 | -4 | 0 | -4 | -8 | -2 | -2 |
| 10 | -4 | 0 | -10 | -15 | -13 | -14 |
| 11 | -54 | 0 | -11 | -16 | -18 | -10 |
| 12 | -7 | 0 | -7 | -2 | -8 | -16 |
| 13 | -20 | 0 | -24 | 0 | -23 | -40 |
| 14 | -32 | 0 | -19 | 0 | -5 | -19 |
| 15 | -23 | 0 | -28 | -19 | -15 | -46 |
| 16 | -17 | -3 | -29 | -21 | -26 | -20 |
| 17 | -2 | 0 | -9 | 0 | 0 | -3 |
| 18 | -2 | 0 | 0 | 0 | -1 | 0 |
| 19 | -9 | 0 | -3 | 0 | -10 | -8 |
| 20 | -14 | 0 | -4 | -3 | -2 | 0 |
| 21 | -34 | 0 | -9 | -4 | -12 | -19 |
| 22 | -7 | 0 | -2 | 0 | -4 | -13 |

Occlusion shear rate

| Condition | 40-Shoulder | 40-Shoulder | 42-Waist | 42-Waist | 40-Waist | 40-Waist |
| --- | --- | --- | --- | --- | --- | --- |
| Time | Pre | Post | Pre | Post | Pre | Post |
| 1 | 9.6 | 34.8 | 18.7 | 12.6 | 22.7 | 10.0 |
| 2 | 11.3 | 50.3 | 5.7 | 6.5 | 11.0 | 2.5 |
| 3 | 7.4 | 8.3 | 26.1 | 15.9 | 9.3 | 8.4 |
| 4 | 41.8 | 51.4 | 34.5 | 64.9 | 49.3 | 33.6 |
| 5 | 6.9 | 23.4 | 20.2 | 14.3 | 16.0 | -14.1 |
| 6 | -7.2 | 85.7 | 34.3 | -2.6 | 6.7 | 14.2 |
| 7 | 29.6 | 75.2 | 35.3 | 18.7 | 19.2 | 14.1 |
| 8 | 13.8 | 37.9 | 17.4 | 10.0 | 4.1 | 8.7 |
| 9 | 29.3 | 51.8 | 17.0 | 26.7 | 21.6 | 20.1 |
| 10 | 74.5 | 50.9 | 45.5 | 40.9 | 14.6 | 14.1 |
| 11 | 12.6 | 16.1 | 20.5 | 26.4 | 5.3 | 15.1 |
| 12 | 7.2 | 77.5 | 20.0 | 20.5 | 30.7 | 3.6 |
| 13 | 19.1 | 37.6 | 24.9 | 12.1 | 10.1 | 3.7 |
| 14 | 5.5 | 21.8 | 9.1 | 14.6 | 11.4 | 7.3 |
| 15 | 16.4 | 51.6 | 31.4 | 18.4 | 22.6 | 17.1 |
| 16 | 11.4 | 31.5 | 18.0 | 1.5 | 15.1 | 11.1 |
| 17 | 9.7 | 27.4 | -3.3 | 8.5 | 13.4 | 1.8 |
| 18 | 3.0 | 37.6 | 13.4 | 32.6 | 10.2 | 5.2 |
| 19 | 15.0 | 34.7 | 19.9 | 24.4 | 15.5 | 20.5 |
| 20 | 33.9 | 51.0 | 10.0 | 40.4 | 32.1 | 15.6 |
| 21 | 15.5 | 89.3 | 9.1 | 42.3 | 38.9 | 15.4 |
| 22 | 61.4 | 70.2 | 37.0 | 65.2 | 8.5 | 17.3 |

Blood flow

| Condition | 40-Shoulder | 40-Shoulder | 42-Waist | 42-Waist | 40-Waist | 40-Waist |
| --- | --- | --- | --- | --- | --- | --- |
| Time | Pre | Post | Pre | Post | Pre | Post |
| 1 | 12.0 | 412.5 | 17.5 | 121.3 | 45.5 | 19.2 |
| 2 | 15.5 | 177.8 | 25.2 | 52.4 | 33.5 | 9.7 |
| 3 | 18.5 | 176.4 | 44.3 | 162.3 | 32.3 | 33.6 |
| 4 | 14.2 | 73.0 | 10.3 | 16.7 | 25.6 | 39.9 |
| 5 | 29.9 | 140.3 | 64.2 | 37.7 | 36.8 | 7.4 |
| 6 | 6.1 | 177.2 | 14.0 | 13.7 | 3.7 | 12.7 |
| 7 | 23.4 | 43.0 | 10.1 | 11.3 | 9.8 | 3.8 |
| 8 | 22.4 | 199.2 | 9.0 | 28.0 | 16.2 | 21.8 |
| 9 | 6.9 | 68.0 | 13.8 | 2.8 | 13.3 | 2.2 |
| 10 | 48.4 | 116.8 | 29.6 | 73.4 | 64.3 | 8.5 |
| 11 | 58.8 | 117.6 | 141.0 | 142.9 | 27.8 | 12.3 |
| 12 | 12.9 | 142.1 | 13.9 | 44.1 | 10.1 | 2.9 |
| 13 | 19.2 | 92.3 | 29.1 | 59.0 | 24.1 | 12.8 |
| 14 | 18.5 | 160.0 | 30.8 | 65.6 | 15.0 | 20.8 |
| 15 | 22.3 | 57.1 | 26.1 | 48.9 | 20.3 | 8.7 |
| 16 | 5.0 | 3.1 | 3.4 | 4.6 | 4.1 | 3.4 |
| 17 | 128.8 | 130.0 | 92.7 | 85.8 | 78.1 | 37.8 |
| 18 | 18.5 | 308.1 | 37.5 | 82.1 | 21.5 | 47.0 |
| 19 | 50.6 | 186.5 | 35.3 | 249.5 | 178.1 | 48.1 |
| 20 | 19.3 | 100.7 | 30.6 | 144.8 | 49.6 | 86.5 |
| 21 | 14.2 | 211.9 | 8.8 | 186.6 | 22.1 | 14.0 |
| 22 | 59.1 | 319.4 | 106.4 | 272.3 | 132.3 | 52.2 |

Occlusion blood flow

| Condition | 40-Shoulder | 40-Shoulder | 42-Waist | 42-Waist | 40-Waist | 40-Waist |
| --- | --- | --- | --- | --- | --- | --- |
| Time | Pre | Post | Pre | Post | Pre | Post |
| 1 | 16.3 | 66.8 | 24.2 | 22.0 | 29.5 | 16.6 |
| 2 | 11.8 | 58.0 | 5.3 | 6.9 | 9.0 | 1.9 |
| 3 | 9.0 | 10.5 | 33.1 | 20.2 | 11.3 | 9.0 |
| 4 | 19.3 | 27.0 | 20.4 | 21.4 | 24.6 | 15.5 |
| 5 | 5.6 | 19.7 | 14.2 | 9.8 | 11.6 | -7.4 |
| 6 | -2.9 | 31.7 | 8.2 | 0.1 | 2.1 | 3.1 |
| 7 | 8.0 | 21.8 | 9.3 | 4.6 | 4.4 | 3.7 |
| 8 | 11.7 | 41.2 | 11.7 | 7.4 | 3.7 | 7.4 |
| 9 | 15.1 | 31.2 | 9.0 | 15.1 | 11.9 | 10.8 |
| 10 | 55.0 | 45.6 | 42.3 | 30.9 | 9.7 | 9.4 |
| 11 | 10.5 | 16.0 | 20.3 | 24.6 | 5.6 | 10.8 |
| 12 | 3.3 | 37.8 | 9.2 | 8.3 | 14.3 | 1.2 |
| 13 | 8.8 | 13.7 | 10.9 | 5.9 | 5.1 | 2.0 |
| 14 | 3.1 | 17.1 | 5.1 | 8.1 | 8.1 | 4.8 |
| 15 | 5.1 | 17.8 | 10.3 | 4.9 | 7.5 | 5.3 |
| 16 | 1.1 | 3.0 | 1.5 | 0.1 | 1.1 | 3.6 |
| 17 | 8.0 | 21.0 | -1.4 | 5.6 | 10.1 | 2.6 |
| 18 | 3.6 | 49.2 | 13.5 | 39.6 | 11.4 | 6.8 |
| 19 | 18.0 | 37.7 | 15.7 | 30.0 | 15.1 | 16.7 |
| 20 | 34.2 | 71.5 | 9.1 | 43.1 | 24.8 | 16.7 |
| 21 | 5.2 | 35.7 | 5.3 | 19.4 | 12.4 | 5.7 |
| 22 | 40.4 | 59.0 | 37.8 | 70.7 | 7.9 | 18.2 |

SRAUC

| Condition | 40-Shoulder | 40-Shoulder | 42-Waist | 42-Waist | 40-Waist | 40-Waist |
| --- | --- | --- | --- | --- | --- | --- |
| Time | Pre | Post | Pre | Post | Pre | Post |
| 1 | 14390 | 32099 | 16834 | 19062 | 17101 | 26235 |
| 2 | 28111 | 34961 | 22063 | 12583 | 28041 | 15448 |
| 3 | 17958 | 23368 | 23146 | 23921 | 17998 | 18793 |
| 4 | 28847 | 24882 | 20301 | 25869 | 24588 | 27032 |
| 5 | 24307 | 33238 | 36734 | 29888 | 37357 | 21520 |
| 6 | 20631 | 24681 | 31855 | 46010 | 35205 | 30117 |
| 7 | 30896 | 42527 | 21549 | 21320 | 23049 | 20225 |
| 8 | 20063 | 30445 | 15690 | 12066 | 14580 | 8449 |
| 9 | 14546 | 30249 | 15364 | 22806 | 19543 | 11307 |
| 10 | 45133 | 27954 | 42920 | 31707 | 31695 | 19017 |
| 11 | 20338 | 30336 | 33893 | 23828 | 21027 | 18503 |
| 12 | 14159 | 47119 | 16939 | 32274 | 19430 | 12097 |
| 13 | 36811 | 47979 | 31263 | 26057 | 29185 | 29301 |
| 14 | 12694 | 22889 | 26740 | 32028 | 13568 | 22020 |
| 15 | 21968 | 21990 | 25765 | 30096 | 20957 | 21313 |
| 16 | 21739 | 34249 | 26622 | 25030 | 24214 | 27523 |
| 17 | 26089 | 31304 | 32559 | 27716 | 30120 | 32749 |
| 18 | 14905 | 23894 | 17942 | 41674 | 20006 | 22036 |
| 19 | 23143 | 43952 | 26225 | 20331 | 20949 | 28458 |
| 20 | 25670 | 23951 | 21595 | 28320 | 33245 | 20707 |
| 21 | 22006 | 40349 | 29531 | 30665 | 32108 | 23198 |
| 22 | 35498 | 36467 | 34681 | 37554 | 26819 | 24549 |

TTP

| Condition | 40-Shoulder | 40-Shoulder | 42-Waist | 42-Waist | 40-Waist | 40-Waist |
| --- | --- | --- | --- | --- | --- | --- |
| Time | Pre | Post | Pre | Post | Pre | Post |
| 1 | 32.636 | 77.427 | 42.102 | 65.183 | 38.006 | 34.353 |
| 2 | 61.681 | 78.953 | 79.025 | 48.652 | 46.989 | 52.25 |
| 3 | 40.728 | 75.47 | 46.082 | 54.515 | 46.718 | 40.811 |
| 4 | 54.711 | 45.225 | 50.336 | 27.065 | 32.495 | 35.45 |
| 5 | 51.709 | 63.641 | 76.284 | 46.965 | 65.806 | 38.233 |
| 6 | 38.273 | 57.985 | 29.844 | 56.324 | 41.626 | 57.438 |
| 7 | 68.146 | 69.92 | 58.026 | 44.051 | 41.174 | 43.219 |
| 8 | 42.315 | 55.909 | 49.469 | 40.787 | 50.15 | 39.897 |
| 9 | 32.719 | 50.089 | 56.505 | 50.965 | 38.36 | 34.69 |
| 10 | 77.028 | 41.281 | 70.035 | 55.558 | 64.047 | 36.792 |
| 11 | 40.492 | 71.455 | 45.035 | 50.947 | 33.986 | 32.524 |
| 12 | 42.777 | 72.457 | 41.982 | 59.284 | 46.306 | 22.341 |
| 13 | 63.316 | 55.031 | 77.13 | 45.179 | 58.179 | 66.55 |
| 14 | 31.372 | 27.144 | 36.088 | 53.362 | 25.348 | 37.108 |
| 15 | 34.979 | 44.69 | 37.351 | 46.824 | 30.627 | 44.029 |
| 16 | 33.299 | 38.411 | 36.914 | 47.036 | 34.075 | 42.81 |
| 17 | 55.143 | 78.173 | 69.255 | 69.41 | 83.541 | 49.437 |
| 18 | 48.691 | 57.555 | 33.547 | 119.12 | 41.033 | 60.919 |
| 19 | 62.066 | 112.752 | 55.744 | 86.2 | 43.828 | 109.94 |
| 20 | 54.337 | 44.509 | 58.356 | 73.503 | 59.298 | 41.718 |
| 21 | 40.17 | 54.685 | 42.308 | 38.247 | 43.526 | 37.592 |
| 22 | 84.974 | 80.662 | 57.105 | 54.516 | 47.558 | 45.982 |

FMD (mm)

| Condition | 40-Shoulder | 40-Shoulder | 42-Waist | 42-Waist | 40-Waist | 40-Waist |
| --- | --- | --- | --- | --- | --- | --- |
| Time | Pre | Post | Pre | Post | Pre | Post |
| 1 | 0.058 | 0.011 | 0.043 | 0.065 | 0.027 | 0.064 |
| 2 | 0.039 | -0.02 | 0.02 | 0.065 | 0.02 | -0.007 |
| 3 | 0.03 | 0.014 | 0.024 | 0.022 | 0.03 | 0.029 |
| 4 | 0.008 | 0.01 | 0.003 | 0.023 | 0.003 | 0.037 |
| 5 | 0.022 | -0.002 | 0.031 | 0.045 | 0.013 | 0.024 |
| 6 | 0.058 | 0.022 | 0.042 | 0.026 | 0.043 | 0.046 |
| 7 | 0.028 | 0.021 | 0.036 | 0.031 | 0.035 | 0.027 |
| 8 | 0.016 | 0.014 | 0.009 | 0.019 | 0.02 | 0.022 |
| 9 | 0.016 | 0.014 | 0.042 | 0.015 | 0.01 | 0.034 |
| 10 | 0.027 | 0.032 | 0.015 | 0.03 | 0.035 | 0.054 |
| 11 | 0.02 | 0.01 | 0.015 | 0.019 | 0.041 | 0.012 |
| 12 | 0.007 | 0 | 0.014 | 0.027 | 0.039 | 0.002 |
| 13 | 0.025 | 0.009 | 0.035 | -0.004 | 0.035 | 0.024 |
| 14 | 0.026 | 0.001 | 0.034 | 0.042 | 0.03 | 0.027 |
| 15 | 0.025 | 0.009 | 0.035 | 0.015 | 0.032 | 0.017 |
| 16 | 0.023 | 0.014 | 0.013 | 0.017 | 0.015 | 0.018 |
| 17 | 0.009 | -0.006 | 0.035 | 0.02 | 0.035 | 0.048 |
| 18 | 0.048 | 0.037 | 0.056 | 0.021 | 0.028 | 0.029 |
| 19 | 0.037 | 0.001 | 0.023 | -0.023 | 0.016 | 0.028 |
| 20 | 0.044 | -0.004 | 0.036 | 0.022 | 0.055 | 0.034 |
| 21 | 0.021 | 0 | 0.051 | 0 | 0.029 | 0.032 |
| 22 | 0.018 | 0.014 | 0.017 | -0.006 | 0.016 | 0.041 |

Unadjusted FMD%

| Condition | 40-Shoulder | 40-Shoulder | 42-Waist | 42-Waist | 40-Waist | 40-Waist |
| --- | --- | --- | --- | --- | --- | --- |
| Time | Pre | Post | Pre | Post | Pre | Post |
| 1 | 11.8 | 1.9 | 9.1 | 13.1 | 5.5 | 13.1 |
| 2 | 9.3 | -3.9 | 5.0 | 15.9 | 5.1 | -1.8 |
| 3 | 6.7 | 3.0 | 5.3 | 4.9 | 6.5 | 6.7 |
| 4 | 2.2 | 2.7 | 0.8 | 7.7 | 0.9 | 11.8 |
| 5 | 5.7 | -0.5 | 8.2 | 12.6 | 3.4 | 6.5 |
| 6 | 17.9 | 6.4 | 16.5 | 10.2 | 17.8 | 18.9 |
| 7 | 9.4 | 6.6 | 12.4 | 10.8 | 11.8 | 10.0 |
| 8 | 3.9 | 3.1 | 2.3 | 4.8 | 5.0 | 5.4 |
| 9 | 4.6 | 3.4 | 12.9 | 4.2 | 2.9 | 9.8 |
| 10 | 6.8 | 7.7 | 3.6 | 7.6 | 8.4 | 15.7 |
| 11 | 4.8 | 2.1 | 3.4 | 4.4 | 10.0 | 3.0 |
| 12 | 2.0 | 0.0 | 4.1 | 8.5 | 12.5 | 0.7 |
| 13 | 7.9 | 2.6 | 11.5 | -1.2 | 11.0 | 7.3 |
| 14 | 7.0 | 0.2 | 9.4 | 11.3 | 7.9 | 7.1 |
| 15 | 8.1 | 2.6 | 11.8 | 5.0 | 10.5 | 5.9 |
| 16 | 11.4 | 6.1 | 6.3 | 8.2 | 8.1 | 6.5 |
| 17 | 2.1 | -1.3 | 8.6 | 4.9 | 8.7 | 12.5 |
| 18 | 10.8 | 7.7 | 12.6 | 4.4 | 6.2 | 6.0 |
| 19 | 8.5 | 0.2 | 5.7 | -4.5 | 3.6 | 6.7 |
| 20 | 10.0 | -0.8 | 8.5 | 4.7 | 14.2 | 7.7 |
| 21 | 6.9 | 0.0 | 15.5 | 0.0 | 9.6 | 10.5 |
| 22 | 4.8 | 3.2 | 3.9 | -1.3 | 3.8 | 9.5 |

Adjusted FMD%

| Condition | 40-Shoulder | 40-Shoulder | 42-Waist | 42-Waist | 40-Waist | 40-Waist |
| --- | --- | --- | --- | --- | --- | --- |
| Time | Pre | Post | Pre | Post | Pre | Post |
| 1 | 14.5 | 5.8 | 11.4 | 16 | 8.1 | 15.9 |
| 2 | 10.5 | -1.2 | 5.7 | 16.8 | 5.6 | -1.3 |
| 3 | 8.5 | 5.2 | 7.2 | 6.7 | 8.5 | 8.1 |
| 4 | 2 | 2.6 | 0.6 | 5.6 | 0.3 | 10.1 |
| 5 | 6.1 | 0.9 | 8.4 | 12.2 | 3.8 | 6.4 |
| 6 | 16.4 | 5.7 | 12.6 | 6.6 | 13.4 | 14.5 |
| 7 | 7.3 | 5.2 | 9.9 | 8.2 | 9.5 | 6.9 |
| 8 | 4.8 | 5 | 2.7 | 5.3 | 5.6 | 6.2 |
| 9 | 3.9 | 4.3 | 11.5 | 3.8 | 2.1 | 9 |
| 10 | 7.5 | 8.8 | 4.8 | 8.2 | 9.5 | 14.9 |
| 11 | 5.8 | 4.3 | 5 | 5.8 | 10.9 | 3.7 |
| 12 | 1.3 | 0.6 | 3.2 | 7 | 10.7 | -1.4 |
| 13 | 6.4 | 2 | 9.5 | -1.8 | 9.5 | 6.1 |
| 14 | 6.9 | 1.5 | 9.1 | 11.3 | 8 | 7.3 |
| 15 | 6.3 | 1.9 | 9.6 | 3.1 | 8.5 | 3.5 |
| 16 | 5.5 | 1.6 | 0.9 | 2.7 | 1.6 | 3.8 |
| 17 | 3.4 | 0.4 | 9.5 | 5.7 | 9.5 | 12.9 |
| 18 | 12.5 | 10.2 | 14.4 | 6.8 | 8 | 8.5 |
| 19 | 10 | 2.5 | 6.5 | -1.8 | 5.2 | 7.8 |
| 20 | 11.7 | 1.7 | 9.7 | 6.8 | 14.6 | 9.3 |
| 21 | 5 | 0.1 | 14.3 | 0 | 7.6 | 8.5 |
| 22 | 4.9 | 4.8 | 5.5 | 0.9 | 5 | 11 |

OIV (mm)

| Condition | 40-Shoulder | 40-Shoulder | 42-Waist | 42-Waist | 40-Waist | 40-Waist |
| --- | --- | --- | --- | --- | --- | --- |
| Time | Pre | Post | Pre | Post | Pre | Post |
| 1 | -0.027 | 0.029 | 0.004 | -0.036 | 0.023 | -0.03 |
| 2 | -0.013 | 0.049 | -0.001 | -0.03 | 0.001 | 0.024 |
| 3 | -0.012 | 0.006 | -0.013 | -0.003 | 0.001 | -0.008 |
| 4 | 0.029 | 0.016 | -0.005 | 0 | 0.006 | -0.023 |
| 5 | -0.002 | 0.025 | -0.001 | -0.018 | 0.003 | 0.002 |
| 6 | -0.017 | 0.033 | -0.014 | 0.012 | 0.005 | -0.01 |
| 7 | 0.012 | 0.03 | 0.016 | 0.02 | 0.037 | -0.009 |
| 8 | 0.002 | 0.01 | 0.015 | 0.011 | -0.008 | -0.007 |
| 9 | -0.001 | 0.043 | -0.026 | 0.005 | -0.015 | -0.009 |
| 10 | 0.007 | -0.004 | -0.003 | -0.003 | 0.037 | -0.035 |
| 11 | 0.006 | 0.033 | 0.012 | 0.012 | -0.013 | 0.018 |
| 12 | 0.011 | 0.051 | 0.006 | -0.005 | -0.03 | 0.012 |
| 13 | -0.013 | 0.036 | -0.013 | 0.027 | -0.016 | 0 |
| 14 | 0.012 | 0.026 | 0 | 0.014 | -0.009 | 0.002 |
| 15 | 0.016 | 0.036 | -0.004 | 0.031 | 0.005 | 0 |
| 16 | 0.002 | 0.027 | 0.015 | 0.006 | 0.004 | -0.012 |
| 17 | 0.024 | 0.055 | 0.017 | 0.026 | 0.006 | -0.011 |
| 18 | -0.006 | 0.003 | 0.005 | 0.012 | 0.002 | 0.024 |
| 19 | -0.029 | 0.027 | 0.003 | 0.04 | 0.006 | 0.008 |
| 20 | 0.001 | 0.001 | -0.002 | 0.019 | -0.011 | -0.005 |
| 21 | 0.007 | 0.053 | -0.027 | 0.042 | 0.006 | -0.004 |
| 22 | -0.002 | 0.033 | 0.004 | 0.028 | 0.002 | -0.012 |

Adjusted OIV

| Condition | 40-Shoulder | 40-Shoulder | 42-Waist | 42-Waist | 40-Waist | 40-Waist |
| --- | --- | --- | --- | --- | --- | --- |
| Time | Pre | Post | Pre | Post | Pre | Post |
| 1 | 4.5 | -6.6 | -1.5 | 6.0 | -5.7 | 5.1 |
| 2 | 2.7 | -11.6 | 0.0 | 6.6 | -0.4 | -6.7 |
| 3 | 2.1 | -1.9 | 2.2 | 0.2 | -0.8 | 1.4 |
| 4 | -8.6 | -4.5 | 1.4 | 0.6 | -1.6 | 7.2 |
| 5 | 0.4 | -6.6 | 0.2 | 4.9 | -0.9 | -0.5 |
| 6 | 5.3 | -10.4 | 6.2 | -3.9 | -0.9 | 5.0 |
| 7 | -3.6 | -10.0 | -5.1 | -6.7 | -13.5 | 4.1 |
| 8 | -0.7 | -2.8 | -4.1 | -3.0 | 1.7 | 1.4 |
| 9 | 0.5 | -12.1 | 7.7 | -1.3 | 4.4 | 2.7 |
| 10 | -2.0 | 0.7 | 0.4 | 0.6 | -10.1 | 9.4 |
| 11 | -1.8 | -8.3 | -3.3 | -3.3 | 2.8 | -4.9 |
| 12 | -3.1 | -15.0 | -1.5 | 2.0 | 9.2 | -3.6 |
| 13 | 4.3 | -11.5 | 4.6 | -8.3 | 5.2 | 0.3 |
| 14 | -3.3 | -6.9 | 0.1 | -3.9 | 2.3 | -0.6 |
| 15 | -5.0 | -11.5 | 1.9 | -10.8 | -1.1 | 0.7 |
| 16 | 0.6 | -12.0 | -6.1 | -1.4 | -0.3 | 4.9 |
| 17 | -6.3 | -14.4 | -4.6 | -7.1 | -1.7 | 2.7 |
| 18 | 0.9 | -1.3 | -1.6 | -3.3 | -1.0 | -6.0 |
| 19 | 5.9 | -6.7 | -1.0 | -9.4 | -1.8 | -2.3 |
| 20 | -0.7 | -1.0 | 0.1 | -4.9 | 2.6 | 0.7 |
| 21 | -1.8 | -16.6 | 7.9 | -12.7 | -1.5 | 1.8 |
| 22 | 0.5 | -8.6 | -1.4 | -7.0 | -0.8 | 2.3 |

Unadjusted OIV

| Condition | 40-Shoulder | 40-Shoulder | 42-Waist | 42-Waist | 40-Waist | 40-Waist |
| --- | --- | --- | --- | --- | --- | --- |
| Time | Pre | Post | Pre | Post | Pre | Post |
| 1 | 5.5 | -5.1 | -0.8 | 7.3 | -4.7 | 6.1 |
| 2 | 3.1 | -9.6 | 0.2 | 7.3 | -0.3 | -6.1 |
| 3 | 2.7 | -1.3 | 2.9 | 0.7 | -0.2 | 1.8 |
| 4 | -7.9 | -4.4 | 1.4 | 0.0 | -1.7 | 7.3 |
| 5 | 0.5 | -5.8 | 0.3 | 5.1 | -0.8 | -0.5 |
| 6 | 5.2 | -9.6 | 5.5 | -4.7 | -2.1 | 4.1 |
| 7 | -4.0 | -9.4 | -5.5 | -7.0 | -12.5 | 3.3 |
| 8 | -0.5 | -2.2 | -3.8 | -2.8 | 2.0 | 1.7 |
| 9 | 0.3 | -10.6 | 8.0 | -1.4 | 4.4 | 2.6 |
| 10 | -1.8 | 1.0 | 0.7 | 0.8 | -8.9 | 10.2 |
| 11 | -1.4 | -7.1 | -2.7 | -2.8 | 3.2 | -4.5 |
| 12 | -3.2 | -12.9 | -1.8 | 1.6 | 9.6 | -4.1 |
| 13 | 4.1 | -10.5 | 4.3 | -7.8 | 5.0 | 0.0 |
| 14 | -3.2 | -6.1 | 0.0 | -3.8 | 2.4 | -0.5 |
| 15 | -5.2 | -10.5 | 1.3 | -10.2 | -1.6 | 0.0 |
| 16 | -1.0 | -11.8 | -7.2 | -2.9 | -2.2 | 4.3 |
| 17 | -5.6 | -12.1 | -4.2 | -6.4 | -1.5 | 2.9 |
| 18 | 1.4 | -0.6 | -1.1 | -2.5 | -0.4 | -5.0 |
| 19 | 6.7 | -5.7 | -0.7 | -7.9 | -1.4 | -1.9 |
| 20 | -0.2 | -0.2 | 0.5 | -4.1 | 2.8 | 1.1 |
| 21 | -2.3 | -14.2 | 8.2 | -11.3 | -2.0 | 1.3 |
| 22 | 0.5 | -7.4 | -0.9 | -5.9 | -0.5 | 2.8 |

FMDDocc (mm)

| Condition | 40-Shoulder | 40-Shoulder | 42-Waist | 42-Waist | 40-Waist | 40-Waist |
| --- | --- | --- | --- | --- | --- | --- |
| Time | Pre | Post | Pre | Post | Pre | Post |
| 1 | 0.031 | 0.04 | 0.047 | 0.029 | 0.05 | 0.034 |
| 2 | 0.026 | 0.029 | 0.019 | 0.035 | 0.021 | 0.017 |
| 3 | 0.018 | 0.02 | 0.011 | 0.019 | 0.031 | 0.021 |
| 4 | 0.037 | 0.026 | -0.002 | 0.023 | 0.009 | 0.014 |
| 5 | 0.02 | 0.023 | 0.03 | 0.027 | 0.016 | 0.026 |
| 6 | 0.041 | 0.055 | 0.028 | 0.038 | 0.048 | 0.036 |
| 7 | 0.04 | 0.051 | 0.052 | 0.051 | 0.072 | 0.018 |
| 8 | 0.018 | 0.024 | 0.024 | 0.03 | 0.012 | 0.015 |
| 9 | 0.015 | 0.057 | 0.016 | 0.02 | -0.005 | 0.025 |
| 10 | 0.034 | 0.028 | 0.012 | 0.027 | 0.072 | 0.019 |
| 11 | 0.026 | 0.043 | 0.027 | 0.031 | 0.028 | 0.03 |
| 12 | 0.018 | 0.051 | 0.02 | 0.022 | 0.009 | 0.014 |
| 13 | 0.012 | 0.045 | 0.022 | 0.023 | 0.019 | 0.024 |
| 14 | 0.038 | 0.027 | 0.034 | 0.056 | 0.021 | 0.029 |
| 15 | 0.041 | 0.045 | 0.031 | 0.046 | 0.037 | 0.017 |
| 16 | 0.025 | 0.041 | 0.028 | 0.023 | 0.019 | 0.006 |
| 17 | 0.033 | 0.049 | 0.052 | 0.046 | 0.041 | 0.037 |
| 18 | 0.042 | 0.04 | 0.061 | 0.033 | 0.03 | 0.053 |
| 19 | 0.008 | 0.028 | 0.026 | 0.017 | 0.022 | 0.036 |
| 20 | 0.045 | -0.003 | 0.034 | 0.041 | 0.044 | 0.029 |
| 21 | 0.028 | 0.053 | 0.024 | 0.042 | 0.035 | 0.028 |
| 22 | 0.016 | 0.047 | 0.021 | 0.022 | 0.018 | 0.029 |

Unadjusted FMDDocc%

| Condition | 40-Shoulder | 40-Shoulder | 42-Waist | 42-Waist | 40-Waist | 40-Waist |
| --- | --- | --- | --- | --- | --- | --- |
| Time | Pre | Post | Pre | Post | Pre | Post |
| 1 | 6.0 | 7.5 | 10.0 | 5.5 | 10.6 | 6.6 |
| 2 | 6.0 | 6.3 | 4.7 | 8.0 | 5.3 | 4.6 |
| 3 | 3.9 | 4.3 | 2.3 | 4.2 | 6.8 | 4.7 |
| 4 | 11.0 | 7.4 | -0.5 | 7.7 | 2.6 | 4.2 |
| 5 | 5.1 | 5.7 | 8.0 | 7.2 | 4.2 | 7.0 |
| 6 | 12.0 | 17.6 | 10.4 | 15.6 | 20.3 | 14.2 |
| 7 | 14.0 | 17.7 | 19.0 | 19.1 | 27.7 | 6.5 |
| 8 | 4.4 | 5.4 | 6.4 | 7.8 | 2.9 | 3.6 |
| 9 | 4.3 | 15.7 | 4.6 | 5.6 | -1.4 | 7.0 |
| 10 | 8.7 | 6.7 | 2.8 | 6.8 | 19.0 | 5.0 |
| 11 | 6.4 | 9.9 | 6.3 | 7.3 | 6.6 | 7.8 |
| 12 | 5.4 | 14.8 | 6.0 | 6.8 | 2.6 | 4.9 |
| 13 | 3.6 | 14.6 | 6.9 | 7.3 | 5.7 | 7.3 |
| 14 | 10.5 | 6.7 | 9.4 | 15.6 | 5.4 | 7.7 |
| 15 | 14.1 | 14.7 | 10.3 | 16.9 | 12.3 | 5.9 |
| 16 | 12.5 | 20.4 | 14.6 | 11.4 | 10.4 | 2.1 |
| 17 | 8.1 | 12.3 | 13.3 | 12.1 | 10.4 | 9.4 |
| 18 | 9.3 | 8.3 | 13.9 | 7.1 | 6.7 | 11.5 |
| 19 | 1.7 | 6.2 | 6.5 | 3.6 | 5.1 | 8.8 |
| 20 | 10.3 | -0.6 | 8.0 | 9.2 | 11.0 | 6.5 |
| 21 | 9.5 | 16.6 | 6.7 | 12.7 | 11.8 | 9.1 |
| 22 | 4.3 | 11.5 | 4.8 | 5.0 | 4.3 | 6.5 |

Adjusted FMDDocc%

| Condition | 40-Shoulder | 40-Shoulder | 42-Waist | 42-Waist | 40-Waist | 40-Waist |
| --- | --- | --- | --- | --- | --- | --- |
| Time | Pre | Post | Pre | Post | Pre | Post |
| 1 | 11.8 | 12.6 | 14.3 | 11.2 | 15.0 | 11.5 |
| 2 | 8.2 | 9.0 | 6.3 | 11.7 | 6.1 | 5.4 |
| 3 | 7.6 | 7.7 | 5.8 | 7.2 | 10.5 | 7.8 |
| 4 | 9.5 | 6.8 | -0.2 | 4.8 | 1.8 | 2.9 |
| 5 | 6.0 | 6.6 | 7.8 | 7.2 | 4.2 | 7.4 |
| 6 | 11.3 | 15.2 | 5.6 | 8.6 | 13.0 | 8.5 |
| 7 | 10.0 | 13.2 | 14.7 | 14.5 | 22.2 | 3.0 |
| 8 | 6.2 | 7.9 | 7.5 | 9.6 | 5.3 | 7.0 |
| 9 | 4.3 | 15.2 | 4.7 | 5.3 | -1.4 | 7.8 |
| 10 | 8.7 | 8.4 | 4.0 | 7.6 | 19.1 | 5.9 |
| 11 | 8.2 | 12.1 | 8.0 | 9.5 | 8.9 | 9.0 |
| 12 | 5.0 | 12.8 | 5.3 | 4.7 | 2.0 | 2.5 |
| 13 | 1.7 | 11.0 | 4.6 | 5.2 | 4.2 | 5.5 |
| 14 | 11.4 | 8.2 | 9.1 | 14.9 | 7.2 | 8.3 |
| 15 | 10.9 | 12.2 | 7.4 | 12.1 | 9.7 | 2.9 |
| 16 | 4.1 | 10.7 | 5.1 | 2.9 | 0.7 | -1.1 |
| 17 | 9.5 | 13.2 | 13.8 | 12.5 | 11.2 | 10.0 |
| 18 | 13.2 | 12.3 | 17.2 | 9.7 | 9.9 | 15.1 |
| 19 | 5.0 | 8.3 | 7.7 | 7.4 | 7.7 | 10.2 |
| 20 | 12.9 | 3.3 | 10.3 | 11.8 | 11.8 | 9.6 |
| 21 | 6.6 | 13.7 | 6.1 | 10.8 | 8.3 | 6.7 |
| 22 | 4.1 | 12.5 | 6.7 | 7.0 | 6.0 | 9.3 |

Diameter

| Condition | 40-Shoulder | 40-Shoulder | 40-Shoulder | 40-Shoulder | 40-Shoulder | 40-Shoulder |
| --- | --- | --- | --- | --- | --- | --- |
| Time | Dbase_pre | Docc_pre | Dpeak_pre | Dbase_post | Docc_post | Dpeak_post |
| 1 | 0.491 | 0.518 | 0.549 | 0.565 | 0.536 | 0.576 |
| 2 | 0.42 | 0.433 | 0.459 | 0.509 | 0.46 | 0.489 |
| 3 | 0.45 | 0.462 | 0.48 | 0.471 | 0.465 | 0.485 |
| 4 | 0.366 | 0.337 | 0.374 | 0.367 | 0.351 | 0.377 |
| 5 | 0.388 | 0.39 | 0.41 | 0.43 | 0.405 | 0.428 |
| 6 | 0.324 | 0.341 | 0.382 | 0.345 | 0.312 | 0.367 |
| 7 | 0.297 | 0.285 | 0.325 | 0.318 | 0.288 | 0.339 |
| 8 | 0.408 | 0.406 | 0.424 | 0.457 | 0.447 | 0.471 |
| 9 | 0.345 | 0.346 | 0.361 | 0.406 | 0.363 | 0.42 |
| 10 | 0.398 | 0.391 | 0.425 | 0.414 | 0.418 | 0.446 |
| 11 | 0.414 | 0.408 | 0.434 | 0.467 | 0.434 | 0.477 |
| 12 | 0.346 | 0.335 | 0.353 | 0.395 | 0.344 | 0.395 |
| 13 | 0.318 | 0.331 | 0.343 | 0.344 | 0.308 | 0.353 |
| 14 | 0.374 | 0.362 | 0.4 | 0.429 | 0.403 | 0.43 |
| 15 | 0.307 | 0.291 | 0.332 | 0.343 | 0.307 | 0.352 |
| 16 | 0.202 | 0.2 | 0.225 | 0.228 | 0.201 | 0.242 |
| 17 | 0.429 | 0.405 | 0.438 | 0.453 | 0.398 | 0.447 |
| 18 | 0.444 | 0.45 | 0.492 | 0.483 | 0.48 | 0.52 |
| 19 | 0.434 | 0.463 | 0.471 | 0.477 | 0.45 | 0.478 |
| 20 | 0.44 | 0.439 | 0.484 | 0.491 | 0.49 | 0.487 |
| 21 | 0.303 | 0.296 | 0.324 | 0.373 | 0.32 | 0.373 |
| 22 | 0.374 | 0.376 | 0.392 | 0.443 | 0.41 | 0.457 |

| Condition | 42-Waist | 42-Waist | 42-Waist | 42-Waist | 42-Waist | 42-Waist |
| --- | --- | --- | --- | --- | --- | --- |
| Time | Dbase_pre | Docc_pre | Dpeak_pre | Dbase_post | Docc_post | Dpeak_post |
| 1 | 0.475 | 0.471 | 0.518 | 0.496 | 0.532 | 0.561 |
| 2 | 0.404 | 0.405 | 0.424 | 0.41 | 0.44 | 0.475 |
| 3 | 0.456 | 0.469 | 0.48 | 0.449 | 0.452 | 0.471 |
| 4 | 0.362 | 0.367 | 0.365 | 0.3 | 0.3 | 0.323 |
| 5 | 0.376 | 0.377 | 0.407 | 0.356 | 0.374 | 0.401 |
| 6 | 0.255 | 0.269 | 0.297 | 0.256 | 0.244 | 0.282 |
| 7 | 0.29 | 0.274 | 0.326 | 0.287 | 0.267 | 0.318 |
| 8 | 0.392 | 0.377 | 0.401 | 0.394 | 0.383 | 0.413 |
| 9 | 0.325 | 0.351 | 0.367 | 0.359 | 0.354 | 0.374 |
| 10 | 0.42 | 0.423 | 0.435 | 0.394 | 0.397 | 0.424 |
| 11 | 0.438 | 0.426 | 0.453 | 0.436 | 0.424 | 0.455 |
| 12 | 0.339 | 0.333 | 0.353 | 0.318 | 0.323 | 0.345 |
| 13 | 0.304 | 0.317 | 0.339 | 0.344 | 0.317 | 0.34 |
| 14 | 0.36 | 0.36 | 0.394 | 0.372 | 0.358 | 0.414 |
| 15 | 0.297 | 0.301 | 0.332 | 0.303 | 0.272 | 0.318 |
| 16 | 0.207 | 0.192 | 0.22 | 0.207 | 0.201 | 0.224 |
| 17 | 0.408 | 0.391 | 0.443 | 0.405 | 0.379 | 0.425 |
| 18 | 0.443 | 0.438 | 0.499 | 0.477 | 0.465 | 0.498 |
| 19 | 0.404 | 0.401 | 0.427 | 0.509 | 0.469 | 0.486 |
| 20 | 0.422 | 0.424 | 0.458 | 0.464 | 0.445 | 0.486 |
| 21 | 0.329 | 0.356 | 0.38 | 0.372 | 0.33 | 0.372 |
| 22 | 0.44 | 0.436 | 0.457 | 0.471 | 0.443 | 0.465 |

| Condition | 40-Waist | 40-Waist | 40-Waist | 40-Waist | 40-Waist | 40-Waist |
| --- | --- | --- | --- | --- | --- | --- |
| Time | Dbase_pre | Docc_pre | Dpeak_pre | Dbase_post | Docc_post | Dpeak_post |
| 1 | 0.494 | 0.471 | 0.521 | 0.488 | 0.518 | 0.552 |
| 2 | 0.394 | 0.393 | 0.414 | 0.393 | 0.369 | 0.386 |
| 3 | 0.459 | 0.458 | 0.489 | 0.435 | 0.443 | 0.464 |
| 4 | 0.351 | 0.345 | 0.354 | 0.314 | 0.337 | 0.351 |
| 5 | 0.388 | 0.385 | 0.401 | 0.372 | 0.37 | 0.396 |
| 6 | 0.241 | 0.236 | 0.284 | 0.244 | 0.254 | 0.29 |
| 7 | 0.297 | 0.26 | 0.332 | 0.27 | 0.279 | 0.297 |
| 8 | 0.402 | 0.41 | 0.422 | 0.409 | 0.416 | 0.431 |
| 9 | 0.343 | 0.358 | 0.353 | 0.346 | 0.355 | 0.38 |
| 10 | 0.416 | 0.379 | 0.451 | 0.344 | 0.379 | 0.398 |
| 11 | 0.409 | 0.422 | 0.45 | 0.404 | 0.386 | 0.416 |
| 12 | 0.311 | 0.341 | 0.35 | 0.296 | 0.284 | 0.298 |
| 13 | 0.318 | 0.334 | 0.353 | 0.329 | 0.329 | 0.353 |
| 14 | 0.38 | 0.389 | 0.41 | 0.38 | 0.378 | 0.407 |
| 15 | 0.306 | 0.301 | 0.338 | 0.29 | 0.29 | 0.307 |
| 16 | 0.186 | 0.182 | 0.201 | 0.279 | 0.291 | 0.297 |
| 17 | 0.401 | 0.395 | 0.436 | 0.383 | 0.394 | 0.431 |
| 18 | 0.449 | 0.447 | 0.477 | 0.484 | 0.46 | 0.513 |
| 19 | 0.441 | 0.435 | 0.457 | 0.416 | 0.408 | 0.444 |
| 20 | 0.388 | 0.399 | 0.443 | 0.443 | 0.448 | 0.477 |
| 21 | 0.303 | 0.297 | 0.332 | 0.304 | 0.308 | 0.336 |
| 22 | 0.421 | 0.419 | 0.437 | 0.431 | 0.443 | 0.472 |
